# Alternatively spliced isoforms reveal a novel type of PTB domain in CCM2 protein

**DOI:** 10.1038/s41598-019-52386-0

**Published:** 2019-11-01

**Authors:** Xiaoting Jiang, Akhil Padarti, Yanchun Qu, Shen Sheng, Johnathan Abou-Fadel, Ahmed Badr, Jun Zhang

**Affiliations:** Department of Molecular and Translational Medicine (MTM), Texas Tech University Health Science Center El Paso, El Paso, TX 79905 USA

**Keywords:** Gene expression profiling, Comparative genomics, Stroke, Genetics of the nervous system

## Abstract

Cerebral cavernous malformations (CCMs) is a microvascular disorder in the central nervous system. Despite tremendous efforts, the causal genetic mutation in some CCM patients has not be identified, raising the possibility of an unknown CCM locus. The *CCM2/MGC46*0*7* gene has been identified as one of three known genes causing CCMs. In this report, we defined a total of 29 novel exons and 4 novel promoters in CCM2 genomic structure and subsequently identified a total of 50 new alternative spliced isoforms of CCM2 which eventually generated 22 novel protein isoforms. Genetic analysis of CCM2 isoforms revealed that the CCM2 isoforms can be classified into two groups based on their alternative promoters and alternative start codon exons. Our data demonstrated that CCM2 isoforms not only are specific in their subcellular compartmentation but also have distinct cellular expression patterns among various tissues and cells, indicating the pleiotropic cellular roles of CCM2 through their multiple isoforms. In fact, the complexity of the CCM2 genomic structure was reflected by the multiple layers of regulation of CCM2 expression patterns. At the transcriptional level, it is accomplished by alternative promoters, alternative splicing, and multiple transcriptional start sites and termination sites; while at the translational level, it is carried out with various cellular functions with a distinguishable CCM2 protein group pattern, specified abundance and composition of selective isoforms in a cell and tissue specific fashion. Through experimentation, we discovered a unique phosphotyrosine binding (PTB) domain, namely atypical phosphotyrosine binding (aPTB) domain. Some long CCM2 isoform proteins contain both classes of PTB domains, making them a dual PTB domain-containing protein. Both CCM1 and CCM3 can bind competitively to this aPTB domain, indicating CCM2 as the cornerstone for CCM signaling complex (CSC).

## Introduction

Cerebral cavernous malformations (CCMs) are malformed microvasculatures predominantly in the central nervous system, with prevalence in approximately 0.1–0.5% of the population and represent 10% of all vascular malformations^1^. The pathology of CCMs can be characterized by distended capillary cavities enclosed by a thin layer of endothelial cells (EC) lacking the support of intervening parenchyma, making lesions susceptible to stroke. CCMs are located primarily in the brain but can also be found in the other parts of the body such as spinal cord, skin, and rarely the retina. Familial forms of CCM is caused by loss of function mutations in one of three known CCM genes, *KRIT1/CCM1*, *MGC4607/Malcavernin/CCM2*, and *PDCD10/CCM3*. Despite tremendous efforts, there are still CCM patients with mutations that have not yet been defined, suggesting the possible existence of an unknown CCM locus.

CCM2 has a strong interaction with both CCM1 and CCM3 proteins, suggesting all three proteins can form a complex, termed CCM signaling complex (CSC), which might be involved in integrin mediated signaling. Subsequently, CSC interacts with other cellular components to mediate intracellular and cell/extracellular matrix (ECM) signaling^2–6^. Both *in-vitro* and *in-vivo* studies showed that through their interaction^7^, CCM proteins influence the angiogenic performance of vascular ECs by regulating β1-integrin-mediated signaling cascades^8^. Obstructed microvasculature in CCM1 and CCM2 (Ccm1/2) animal models^5^ is the provenance of various phenotypic expressions such as enlarged heart^9^, dilated axial primitive vessels^10,11^, and blood stasis in and around the heart, thus demonstrating the importance of CSC complex in angiogenesis^5,12,13^.

In this study, we aimed to define the CCM2 gene, in both transcriptional and translational levels. We found that there are multiple alternative promoters, alternative splicing, and multiple transcriptional start sites and termination sites in the genomic structure of CCM2 gene, which play significant roles during the transcriptional events, generating various CCM2 RNA isoform species with apparently distinct biological functions. These CCM2 isoforms were further confirmed at the protein level, leading us to identify a novel PTB domain in CCM2 protein, which helps us better understand the complexity of CSC and its associated cellular factors which contribute to the angiogenic events and underlining molecular and cellular etiology in the pathogenesis of CCMs.

## Results

### Identification of new exons, alternative spliced exons, and new isoforms in CCM2

**Figure 1:**
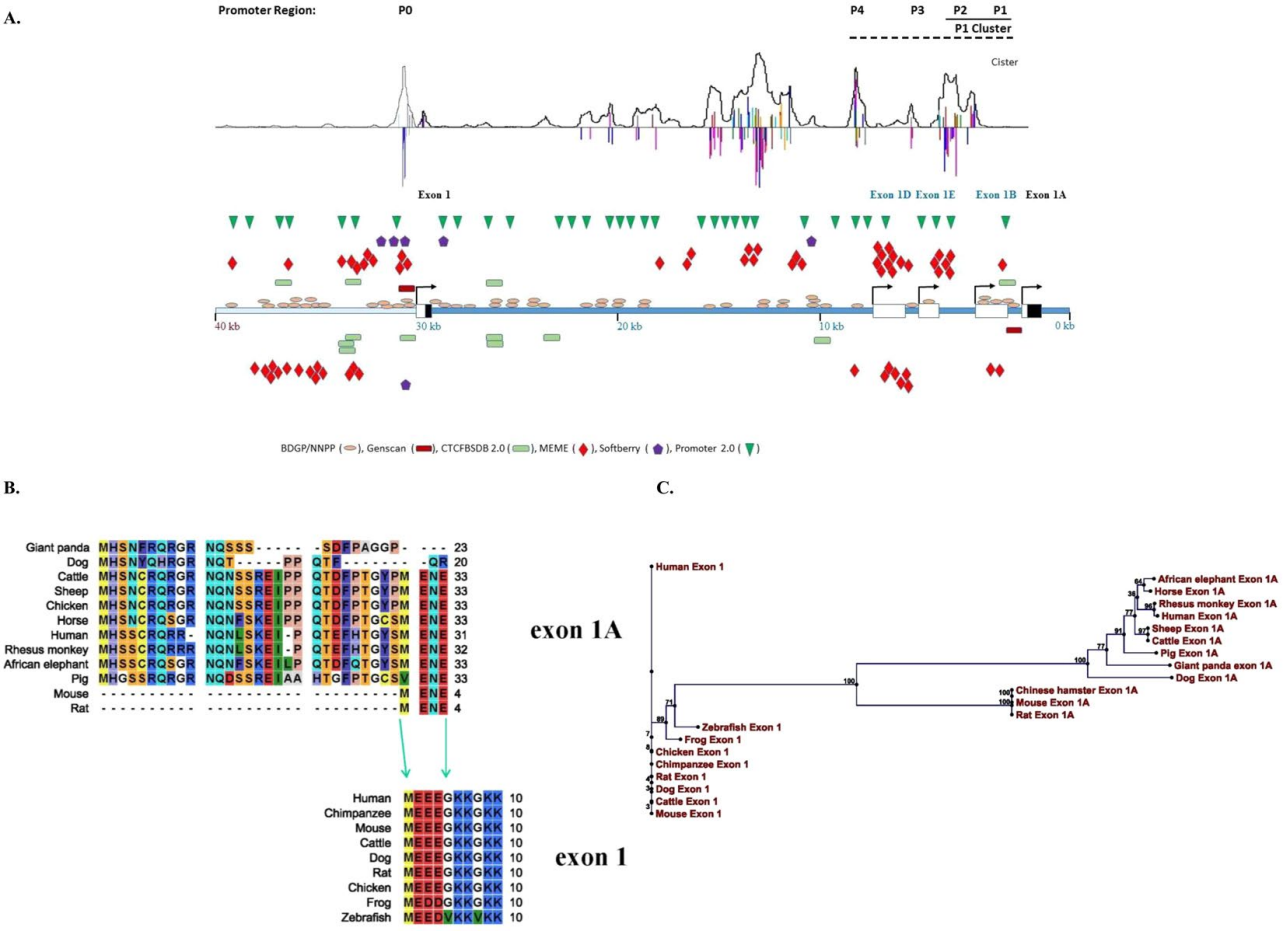
Genomic structure, conservation, and variability among alterative start-codon exons and promotors of CCM2. (**A**) The complex promoter regions of human CCM2 locus were defined with bioinformatics (promoter prediction software from top to bottom: Cister, promotor2.0, Softberry, MEME, CTCFBSDB, BDGP/NNPP and Genscan as indicated by different colors). Symbols on top of DNA templates are on positive strand, below are on negative strand. The single promoter for the original *bonafide* start-codon exon, exon 1, simply lies immediately upstream of the transcription start site for exon 1, as P0. The promoter region for exon 1A is much more complicated. Although a seemingly weak promoter, P1 lies immediately upstream of its transcription-start site; in addition, there are three relatively strong promoters (P2-P4) upstream adjacent to P1 promoter. Three exons (exon 1B, 1D, 1E) with the transcription start site driven by these three promoters (P2-P4), respectively, usually skip exon 1A (coding exon), presumably to down-regulate the transcription level of group B CCM2 isoforms. Genomic structure of 5′ region of the human CCM2 locus is schematically summarized in this map. Noncoding region within a transcription-start exon labeled as white box while coding region within the exon labeled as black box. (**B**) Multiple-alignment between two alterative start codon exons (exon 1 and exon 1A) across species reveals a vertebrate-specific exon 1 and a mammalian-specific exon 1A and their evolutional relationship. Exon 1A is evolutionarily evolved from exon1 with its C-terminus homolog to the N-terminus of exon1. (**C**) Phylogenetic relationships between exon 1A and exon 1 among CCM2 isoforms across species based on neighbor joining (NJ) method which hypothesizes a stochastic process in different lineages during evolution.

**Figure 2:**
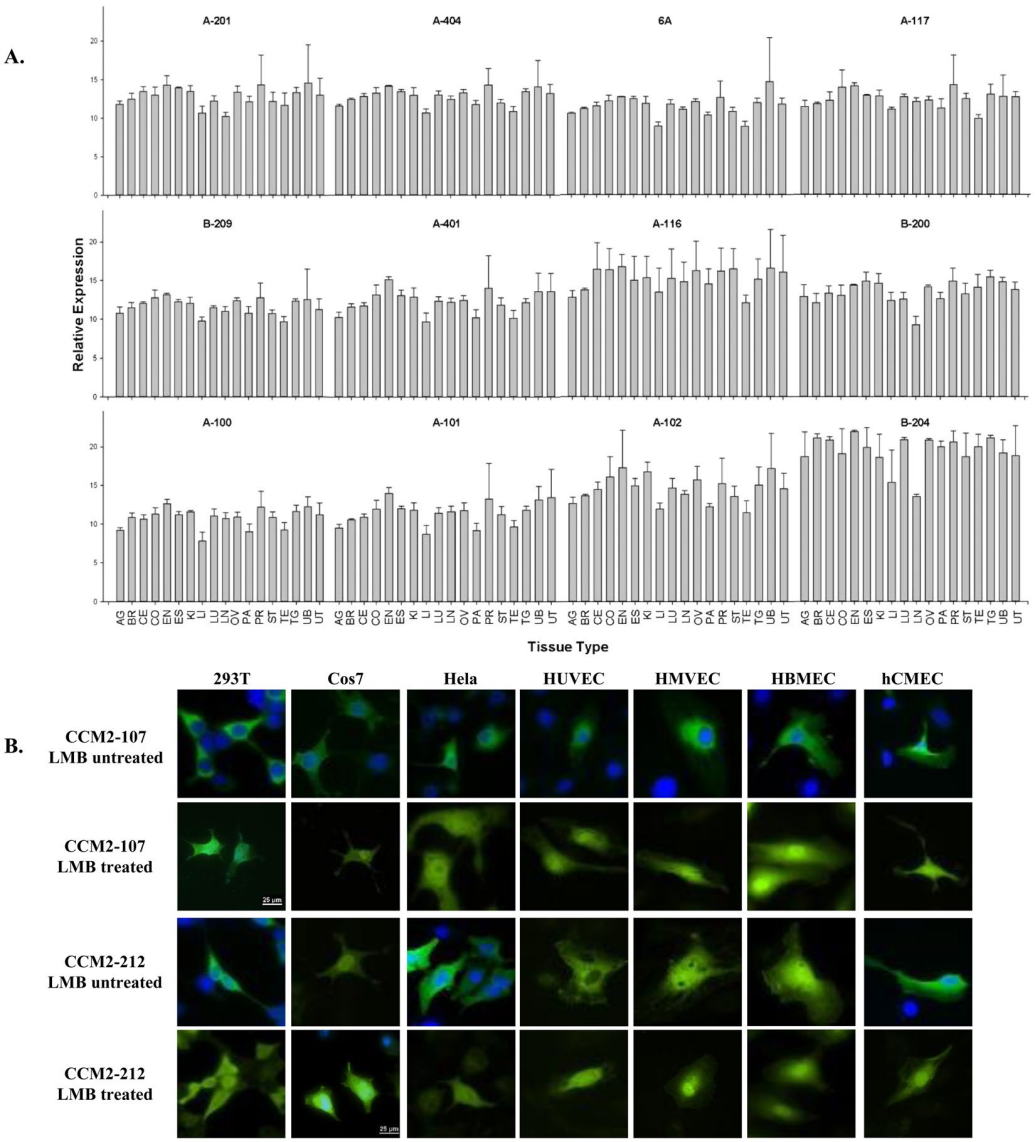
Relative expression profiling of endogenous CCM2 isoforms among various tissues. (**A**) The relative expression (2^−∆CT^) of CCM2 isoforms measured by qPCR in various tissues is presented with bar plots. Allele-specific real-time quantitative PCR (qPCR) assays were performed in triplicates (n = 3) and represented with means and standard deviations (M ± SD) of the relative expressions. Adrenal gland (AG), Breast (BR), Cervix (CE), Colon (CO), Endometrium (EN), Esophagus (ES), kidney (KI), Liver (LI), Lung (LU), Lymph node (LN), Ovary (OV), Pancreas (PA), Prostate (PR), Stomach (ST), Testis (TE), Thyroid gland (TG), Urinary bladder (UB), and Uterus (UT). (**B**) Subcellular localization of CCM2 isoform pairs, CCM2-107 and CCM2-212, in various immortalized cell lines: Immortalized Human Embryonic Kidney cells (293 T), Immortalizing Monkey Kidney Fibroblast cells (COS7), Immortalized Human Cervical Cancer cells (HeLa); and several primary/immortalized human endothelial cell lines: Human Umbilical Vein Endothelial Cells (HUVEC), Human Microvascular Endothelial Cells (HMVEC), Human Brain Microvascular Endothelial Cells (HBMEC), and Immortalized Human Cerebral Microvascular Endothelial Cells, hCMEC/D3 cells (hCMEC). Isoform CCM2-107s is seen predominantly in the cytoplasm in every cell line but accumulates in the nucleus after treatment with leptomycin (LMB treated). However, isoform CCM2-212 behaves differently and is seen predominantly in the cytoplasm in some cells, but distributed evenly in both the nucleus and cytoplasm before leptomycin treatment (LMB untreated), indicating its differentiated cellular compartmentation, possible nuclear function, and potential association with new cellular partners. Scale bars represent 25 µm and are located in CCM2-107 LMB treated 293 T panel and CCM2-212 LMB treated Cos7 panel; all images were acquired using the same microscope and magnitude and processed identically to each other.

A total of 31 exons, including 8 new exons and 13 new alternative spliced exons derived from existing exons, were identified in the *CCM2* gene. Most of the newly identified exons were located near or at the 5′end, just downstream of the original *bonafide* start codon exon (exon1); as alternative transcription start exons with their own promoters, only two of them (6A, 6B) resided in the middle of CCM2 genomic structure (Table 1, Fig. 1A). Interestingly among newly identified alternative transcription start exons, only one was found harboring another start codon, which makes it a novel alternative start codon exon (exon1A) with its own distinct promoter (Fig. 1A). We then identified a total of 50 isoforms of CCM2 using two sets of full-length *CCM2* gene primers with genomic analysis tools. In addition, 11 new isoforms without exon1 or exon1A were identified. Almost all of these new exons and partial sequences of isoforms have been reported in NCBI EST/ExAC databases, reaffirming their cellular existence (Tables 1, 2). These new CCM2 isoforms are categorized into three groups based on their alternative promoters and alternative start codon exons; A group has a start codon in exon1 with the original promoter (P0) and B group in exon1A with a novel promoter (P1) downstream of exon 1, while C group in other exons are uncertain (Table 2, Fig. 1A). Both A group and B group have a notable biological significance based on their cellular abundance in various tissues (Figs 2A, 4A, Suppl. Fig. 1). Considering the shared identical open-reading frame (ORF) of some CCM2 isoforms, a total of 32 CCM2 isoforms were eventually confirmed with different coding schemes, indicating the complexity of CCM2 isoform regulation at transcription level (Table 2).

**Table 1:** Identification of new exons and alternative spliced exons (as) of *CCM2* gene.

Exons	Genomic position			Hits in EST
	Start	End	Length(bp)	Database (%)
exon 1*	45039933	45039962	30	89
**exon 1A**	***45067304***	***45067396***	***93***	***61***
**exon 1B**	***45066608***	***45066865***	***258***	***5***
**exon 1Bas1**	***45066608***	***45066869***	***262***	***2***
**exon 1Bas2**	***45066608***	***45066821***	***214***	***2***
**exon 1C**	***45069043***	***45069108***	***66***	***2***
**exon 1D**	***45064095***	***45064371***	***277***	***2***
exon 2	**45077852**	**45078025**	174	100
**exon 2as1**	***45077852***	***45078019***	***168***	
**exon 2A**	***45099802***	***45099907***	***106***	***2***
**exon 2B**	***45102692***	***45102814***	***123***	*0*
exon 3	45103517	45103600	84	100
exon 4	45104062	45104245	184	100
**exon 4as1**	***45104062***	***45104097***	***36***	
**exon 4as2**	***45104062***	***45104151***	***90***	
exon 5	45108042	45108178	137	100
exon 6	45109425	45109560	136	100
**exon 6A**	***45111349***	***45111471***	***123***	***13***
**exon 6B**	***45109988***	***45110129***	**142**	***2***
exon 7	45112325	45112382	58	100
**exon 7as1**	45112325	45112356	32	
exon 8	45113059	45113170	112	100
exon 9	45113869	45114007	139	100
**exon 9as1**	***45113841***	***45114007***	***167***	***1***
exon 10**	45115376	45115656	281	100
**exon 10as1**	***45115500***	***45115757***	***258***	
**exon 10as2**	***45115573***	***45115757***	***185***	
**exon 10as3**	***45115721***	***45115757***	***37***	
**exon 10as4**	***45115376***	***45115440***	***65***	100
**exon 10as5**	***45115652***	***45116068***	***417***	100
**exon 10as6**	***45115432***	***45115757***	***326***	100

Genomic location of exons and alternative slicing sites of CCM2 based on the Genome Reference Consortium Human Genome Build 37/Human Genome Assembly 19 (GRCh37/hg19). Both underlined exons 1 and 1A contain a *bonafide* start codon with different promoters. Newly identified exons are highlighted with bold letter, whereas the alternatively spliced exons were italicized (as). The genomic location of initial and end sites and exact length of each exon are detailed in the table. The maximum number of blast hit for each exon in human EST database is set at 100, reaching this limit is considered as the most abundant in CCM2 cDNA/EST, while some blanks in this category indicate that the alterative spliced isoform are located within its coordinated exons. Exon 1 is the foremost 5′ end among all exons along the genomic sequence, *or **indicates exons containing alternative transcriptional start sites; ***containing the alternative termination sites.

**Table 2:** Identification and nomenclature of alternatively spliced CCM2 isoforms.

Isoform identifier	Containing exons	Missing exons	Peptide Length (aa)	cDNA/EST Clones
**CCM2-A**				
CCM2-100	1, 2, 3, 4, 5, 6, 7, 8, 9, 10		444	BX337677
CCM2-101	1, 3, 4, 5, 6, 7, 8, 9, 10	2	386	BM922546
CCM2-102	1, 3, 4, 7, 8, 9, 10	2, 5, 6	295	
CCM2-103	1, 2, 8, 9, 10	3, 4, 5, 6, 7	142*	
CCM2-104	1, 2, 7, 8, 9, 10	3, 4, 5, 6	142*	
CCM2-105	1, 2, 3, 4, 6, 7, 8, 9, 10	5	142*	
CCM2-106	1, 4, 5, 6, 7, 8, 9, 10	2, 3	358	
CCM2-107	1, 6, 7, 8, 9, 10	2, 3, 4, 5	251	
CCM2-108	1, 7, 8, 9, 10	2, 3, 4, 5, 6	142*	
*CCM2-1209*	1, 8, 9, 10	2, 3, 4, 5, 6, 7	*142*	
CCM2-111	1, 5, 6, 7, 8, 9, 10	2, 3, 4	142*	
CCM2-115	1, 2, 5, 6, 7, 8, 9, 10	3, 4	142*	BG385641
CCM2-116	1, 2, 6, 7, 8, 9, 10	3, 4, 5	309	BI752347
CCM2-117	1, 2, 3, 4, 7, 8, 9, 10	5, 6	353	BG421932
CCM2-118	1, 2, 3, 4, 5, 7, 8, 9, 10	6	207	BX439329
CCM2-119	1, 2, 3, 4, 5, 6, 8, 9, 10	7	142*	BX462850
CCM2-120	1, 3, 4, 6, 7, 8, 9, 10	2, 5	142*	CX872822
CCM2-129	1, 7, 8, 9, 10	2, 3, 4, 5, 6	142*	
CCM2-130	1, 1C, 2, 3, 4, 6, 7, 8, 9, 10	5	142*	BX460017
CCM2-131	1, 1C, 2, 7, 8, 9, 10	3, 4, 5, 6	142*	
CCM2-132	1, 1C, 2, 5, 6, 7, 8, 9, 10	3, 4	142*	
CCM2-300	1, 2B, 3, 4, 5, 6, 7, 8, 9, 10	2	142*	BI523801
CCM2-401	1, 3, 4as1, 10as1	2, 5, 6, 7, 8, 9	81	
CCM2-402	1, 2, 3, 4as2, 10as2	5, 6, 7, 8, 9	153	
CCM2-403	1, 2as1, 10as3	3, 4, 5, 6, 7, 8, 9	95	
CCM2-404	1, 2, 3, 4, 7, 8, 9, 10as4, 10as5	5, 6	334	H7C6075-68
CCM2-600	1, 2, 3, 4, 5, 6, 6A, 7, 8, 9, 10		485	BG722235
CCM2-601	1, 3, 4, 5, 6, 6A, 7, 8, 9, 10	2	427	CF145136
CCM2-602	1, 2, 3, 4, 6, 6A, 7, 8, 9, 10	5	142*	
CCM2-603	1, 3, 4, 6, 6A, 7, 8, 9, 10	2, 5	142*	H7C6075-63
CCM2-604	1, 5, 6, 6A, 7, 8, 9, 10	2, 3, 4	142*	CF145056
CCM2-606	1, 4, 5, 6, 6A, 7, 8, 9, 10	2, 3	400	
CCM2-607	1, 6, 6A, 7, 8, 9, 10	2, 3, 4, 5	293	
CCM2-610	1, 2, 3, 4, 5, 6, 6B, 8, 9, 10	7	473	
**CCM2-B**				
CCM2-200	1A, 2, 3, 4, 5, 6, 7, 8, 9, 10	1	465	BX363852
CCM2-201	1A, 3, 4, 5, 6, 7, 8, 9, 10	2	407	BI766123
CCM2-202	1A, 3, 4, 5, 8, 9, 10	2, 6, 7	148	BI906707
CCM2-203	1A, 2, 3, 4, 7, 8, 9, 10	5, 6	374	H7C6075-81
CCM2-204	1A, 2, 4, 5, 6, 7, 8, 9, 10	3	437	H7C6075-86
CCM2-205	1A, 7, 8, 9, 10	2, 3, 4, 5, 6	142*	
CCM2-206	1A, 2, 3, 4, 5, 6, 6A, 7, 8, 9, 10	1	506	BQ073060
CCM2-207	1A, 2, 3, 5, 6, 6B, 8, 9, 10	7	494	BX415190
CCM2-208	1A, 2, 7, 8, 9, 10	3, 4, 5, 6	142*	
CCM2-209	1A, 8, 9, 10	2, 3, 4, 5, 6, 7	178	
CCM2-210	1A, 3, 4, 7, 8, 9, 10	2, 5, 6	316	
CCM2-211	1A, 5, 6, 7, 8, 9, 10	2, 3, 4	142*	
CCM2-212	1A, 6, 7, 8, 9, 10	2, 3, 4, 5	272	
CCM2-213	1A, 2, 3, 4, 5, 6, 7as1, 10as4	8, 9	354	
CCM2-214	1A, 3, 4, 5, 6, 7as1, 10as4	2, 8, 9	296	
CCM2-215	1A, 3, 4, 7as1, 10as4	2, 5, 6, 8, 9	205	
CCM2-216	1A, 2, 3, 4, 6A, 7, 8, 9, 10	5, 6	415	
**CCM2-C**				
CCM2-220	1B, 3, 4, 5, 6, 8, 9, 10	2, 7	142*	
CCM2-221	1B, 2, 8, 9, 10	3, 4, 5, 6, 7	142*	
CCM2-222	1B, 3, 4, 5, 6, 7, 8, 9, 10	2	142*	
CCM2-223	1Bas2, 3, 8, 9, 10	2, 4, 5, 6, 7	179	BI907152
CCM2-224	1B, 7, 8, 9, 10	2, 3, 4, 5, 6	142*	
CCM2-225	1B, 2, 7, 8, 9, 10	3, 4, 5, 6	142*	
CCM2-226	1B, 5, 6, 7, 8, 9, 10	2, 3, 4	142*	
CCM2-227	1B, 1A, 5, 6, 8, 9, 10	2, 3, 4	142*	
CCM2-230	1D, 2, 3, 4, 5, 6, 7, 8, 9, 10		142*	CV572216
CCM2-240	1B, 2, 3, 4, 5, 6, 7, 8, 9, 10		142*	DA983503
CCM2-241	1B, 6, 7, 8, 9, 10	2, 3, 4, 5	142*	

Novel CCM2 isoforms identified can be further validated through EST database search. A specific isoform identifier (IsoID) is assigned for each isoform based on the information of the exon composition. CCM2 isoforms are classified into three major groups based on their alternative promoters and alternative start codon exons: CCM2-A (exon1), CCM2-B (exon1A), and CCM2-C (Undetermined). Among identified isoforms, CCM2-100 isoform is original “*bonafide” CCM2 with* ten coding exons, while *CCM2-1209* isoform is a short protein isoform with a common C-terminal peptide sequence of HH domain (Harmonin homology) shared by many isoforms (such as 109, 209 etc). The genomic structure of the representative isoforms of CCM2 is further validated by their existing correlative cDNA/EST clones in NCBI database. *At peptide length column indicates the isoform sharing the identical open-reading frame with isoform *CCM2-1209* with various length of 5′ UTRs.

### Newly identified CCM2 isoforms are detected at both transcriptional and translational levels

#### Bioinformatics data indicate the pleiotropic roles of CCM2 isoforms

All CCM2 isoforms were analyzed and their functional domains were defined by NCBI conserved domains database using genomic analysis tools (Suppl. Table 3). Noteworthy, CCM2 protein isoforms share identical or similar amino acid sequences like isoforms in other genes and many of them contain a phosphotyrosine binding (PTB) domain which has already been shown to interact with the NPXY motifs (Asn-Pro-X-Tyr, X can be any amino acid) of CCM1 and β1-integrin^7,14^. However, the classic PTB domain was notably missing in several alternatively spliced isoforms (termed PTB-less isoforms). Furthermore, 8 additional different domains with distinct cellular functions were identified across different isoforms (Suppl. Table 3), indicating the pleiotropic roles of these alternatively spliced isoforms in various cellular events. Several new exons with transcription start sites and their own promoters (P0-P5) were identified, but only two of them contain distinct start-codon exons (exons 1 and 1A, labeled with a black box, respectively, Fig. 1A) and are regulated by their alternative promoters, P0 or P1, respectively. Transcripts starting with other exons (exon 1B, 1D, and 1E) usually generate a coding fragment of the canonical CCM2-1209 isoform, which is a common truncated C-terminal fragment containing solely the Harmonin homology (HH) domain. Our preliminary data suggest that promoter P1 might coordinate with P2 (exon 1B) to form a P1 promoter cluster (P1c) for regulation at the transcription level of B group CCM2 isoforms; additionally P3-P5 promoters may also have some influence on this P1c (Fig. 1A), indicating that the transcriptional regulation of B group CCM2 isoforms is more complicated than that of A group CCM2 isoforms.

#### Distinct cellular roles of two major groups of CCM2 isoforms

Multiple alignments (Fig. 1B) and phylogenetic analysis for the start-codon exons between two major groups (Fig. 1C) across species, indicated that the originally identified start codon exon 1 is highly conserved and vertebrate-specific, while the newly identified start codon exon 1A is mammalian-specific. Their evolutionary relationship indicates the mammalian-specific and more variable exon 1A likely originated from more conserved exon 1, probably through a series of genomic recombination and duplication events, based on the homologous sequence of C-terminus of exon1A to the N-terminus of exon 1 (Fig. 1B,C).

The variable nature of exon 1A was shown by the significantly shortened amino acid sequence in rodents (resulting from a 13 bp insertion in the coding region possibly during ancient DNA replication events in mouse and rat), which is further supported by coding variation in giant panda, dog, and even human, thus substantiating phylogenetic results (Fig. 1B,C). Further, the promoter region for exon 1A is much more complicated than the promoter for the original exon 1 (Fig. 1A). This finding led us to hypothesize that different isoforms of CCM2 behave differently in the different cell types and might have different functions in various biogenic events.

#### Differentially transcribed isoform RNAs in various tissues and cell lines

To test our hypothesis, we preformed qPCR which demonstrated that all identified CCM2 isoforms were ubiquitously expressed in all major tissues, but their expression levels varied anatomically. However, many CCM2 isoforms seem to be expressed relatively higher in reproductive tissues including Breast (BR), Cervix (CE), Endometrium (EN), Ovary (OV), Prostate (PR), Testis (TE) and Uterus (UT), compared to other tissues (Fig. 2A). Likewise, detailed gene expression profiling on various brain tissues and cell lines was also performed (Suppl. Fig. 1A,B). Upon elucidating additional data, it was observed that the CCM2 isoforms were ubiquitously distributed in various brain tissues and cell lines albeit at considerably different levels of expressions, suggesting that each CCM2 isoform may have a distinct cellular function.

#### Distinct cellular compartmentation for different isoforms

Efforts for a detailed subcellular localization of CCM2 has thus far been unsuccessful^15^, possibly due to the unknown existence of its polymorphic isoforms. To define the cellular localization of CCM2 isoforms, we first selected one isoform pair, CCM2-107 (A group) and CCM2-212 (B group), to investigate their subcellular localization. Surprisingly, we found significantly differentiated cellular localization patterns in various cell lines. CCM2-107 was predominantly found in the cytoplasm in all tested cell lines but accumulated in the nucleus following leptomycin treatment, similar to the isoform CCM2-100 and its shuttle, CCM1, as described before^7,16^. However, in some cell lines isoform CCM2-212 was distributed evenly in both the nucleus and cytoplasm at steady state before leptomycin treatment (Fig. 2B) which indicates that there are either some cellular factors shuttling these isoforms into the nucleus or isoforms themselves have the ability. To confirm this finding, all identified CCM2 isoform pairs were used to screen in HeLa cells (Immortalized Cervical cancer cells) and three primary human endothelial cell lines, Human Umbilical Vein Endothelial Cells (HUVEC), Human Microvascular Endothelial Cells (HMVEC), and Immortalized Human Cerebral Microvascular Endothelial Cells (hCMEC/D3) (Suppl. Fig. 2A–D), and final results were summarized in Table 3. The data from these experiments reaffirmed our previous observation that some CCM2 isoforms localize differently in different cells, probably through their association with different cellular factors or complexes. Intriguingly, there are more CCM2 isoforms retained within the nucleus at steady state in primary endothelial cell lines suggesting important and diverse cellular roles these nuclear CCM2 isoforms might play during vascular angiogenesis (Table 3, Suppl. Fig. 2).

**Table 3:** Differential cellular compartmentations among different CCM2 isoforms in various cell lines.

Cell Type	Isoform	LMB Untreated	LMB Treated	Cell Localization
**LA**	CCM2-107	C	CN	S
	CCM2-100	C	CN	S
	CCM2-101	C	CN	S
	CCM2-102	C	CN	S
	CCM2-117	C	CN	S
	CCM2-116	C	CN	S
	CCM2-402	CN	CN	S-N
	CCM2-601	C	CN	S
	CCM2-206	C	CN	S
	CCM2-212	CN	CN	S-N
	CCM2-201	C	CN	S
	CCM2-203	C	CN	S
	CCM2-210	C	CN	S
	CCM2-216	C	CN	S
**HU**	CCM2-107	C	CN	S
	CCM2-100	C	CN	S
	CCM2-101	CN	CN	S-N
	CCM2-102	C	CN	S
	CCM2-117	C	CN	S
	CCM2-116	C	CN	S
	CCM2-402	CN	CN	S-N
	CCM2-601	C	CN	S
	CCM2-206	C	CN	S
	CCM2-212	CN	CN	S-N
	CCM2-201	C	CN	S
	CCM2-203	C	CN	S
	CCM2-210	C	CN	S
	CCM2-216	C	CN	S
	CCM2-600	C	CN	S
**HE**	CCM2-212	C	CN	S
	CCM2-100	C	CN	S
	CCM2-101	CN	CN	S-N
	CCM2-102	CN	CN	S-N
	CCM2-117	C	CN	S
	CCM2-116	CN	CN	S-N
	CCM2-402	C	C/N	S
	CCM2-601	CN	CN	S-N
	CCM2-206	CN	CN	S-N
	CCM2-212	CN	CN	S-N
	CCM2-201	CN	CN	S-N
	CCM2-203	C	CN	S
	CCM2-210	CN	CN	S-N
	CCM2-216	CN	CN	S-N
**HC**	CCM2-107	C	C/N	S
	CCM2-100	C	C/N	S
	CCM2-101	C	C/N	S
	CCM2-102	C	C/N	S
	CCM2-117	C	C/N	S
	CCM2-116	C	C/N	S
	CCM2-402	C/N	C/N	S-N
	CCM2-601	C/N	C/N	S-N
	CCM2-206	C	C/N	S
	CCM2-212	C	C/N	S
	CCM2-201	C/N	C/N	S-N
	CCM2-203	C	C/N	S
	CCM2-210	C	C/N	S
	CCM2-216	C	C/N	S

Subcellular localization with /without treatment of leptomycin and proposed shuttling scheme are listed in HeLa cell (LA) and 3 endothelial cell lines: HUVEC (HU), human dermal microvascular endothelial cells (HE), and immortalized human brain microvascular endothelial cells (HC). In the table, C: observed in cytoplasm; N: observed in nucleus; CN: observed in both cytoplasm and nucleus; S: shuttle between cytoplasm and nucleus mostly in cytoplasm in the steady state; S-N: shuttle between cytoplasm and nucleus evenly distributed in cytoplasm and nucleus in the steady state.

#### Differentially translated CCM2 isoforms in various cells

**Figure 3:**
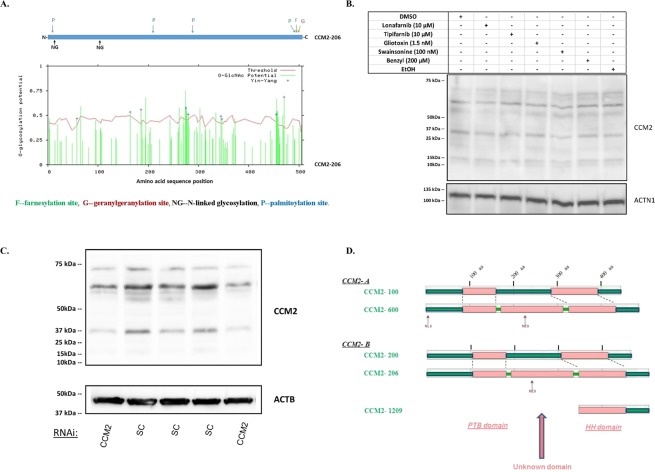
Cellular distribution and motifs/domains among different endogenous CCM2 isoform proteins. (**A**) the potential post-translation modification (PTM) sites were defined with prediction software by searching the longest CCM2 isoform, CCM2-206. In the upper panel, CSS (clustering and scoring strategy) was used to scan palmitoylation sites (P), farnesylation sites (F), and geranylgeranylation sites (G) while NetNGlyc 1.0 was used to predict N-glycosylation sites (NG). In the lower panel, YinOYang 1.2 was used to predict O-glycosylation sites: green bars surpassing the red threshold line have significant chance to be glycosylated at the site through O-glycosylation. Each major PTM is color coded. (**B**) Multiple CCM2 protein bands are not a result of post-translation modifications. Two different vehicle-controls (DMSO, EtOH), inhibitors of farnesylation (Lonafarnib, Tipifarnib, Gliotoxin) and geranylgeranylation (GGTI-298), N-linked glycosylation inhibitor (Swainsonine), and O-linked glycosylation inhibitor (benzyl-α-GalNAc, Benzyl) were used to treat 293 T cells. None of the treatments resulted in missing bands or significantly changed band density, comparable to the controls. (**C**) Multiple CCM2 protein bands are diminished by silencing CCM2. 293 T cells were treated with either CCM2 RNAi (siRNA-CCM2) or scrambled control (SC). Significantly decreased densities of all protein bands of CCM2 were observed consistently (two shown) in CCM2 knockdown cells, relative to SC controls. (**D**) Bioinformatics analysis of potential functional domains and putative linear motifs in CCM2 isoforms. (D1). Two longest isoform pairs of CCM2 from A group (100 and 600) and B group (200 and 206) were selected, to screen for intrinsic globularity with GlobPLot 2.3 and putative linear motifs with ELM, (Eukaryotic linear motifs). Structurally globular regions are considered to be composed of different secondary structures and fold types (pink), in contrast to disorder (unstructured) regions (green). All isoforms of CCM2 from A group and B group share two common globular regions: N-terminal globular region which harbors PTB domain and C-terminal region which covers HH domain (Harmonin homology). Intriguingly, a third globular region was identified, by analyzing two longest isoforms of CCM2, which have an additional newly identified 41 amino acid (aa) peptide coded by exon 6A (CCM2-600 and CCM2-206), compared to their respective paired isoforms, CCM2-100 (**A**) and CCM2-200 (**B**). The appearance of a new middle globular region might suggest an additional secondary structure and fold created in conjunction with this additional peptide. (D2). With motif prediction tool, ELM, we found five major linear protein motifs along CCM2 isoforms. (D2.1). *Motif for protein degradation (red colored)*. 11, signal motif targeting to endoplasmic reticulum (ER) lumen; 12, signal motif targeting the protein for degradation in a cell cycle dependent manner; 13, signal motif targeting the protein for degradation by binding to the UBR-box of N-recognins; 14, S/T rich motif for SPOP/Cul3-dependant ubiquitination; 15, a degron motif, for the cyclin’s degradation; 16, LIR motif in autophagy; 17, di-Arg ER retention motif, targeting to endoplasmic reticulum (ER) lumen; 18, Sorting motif, targeting to the lysosomal-endosomal-complex. (D2.2). *Motif for protein phosphorylation (pink colored)*. 21, canonical motif for the CDK phosphorylation site; 22, canonical motif for MAP kinases docking or phosphorylation site; 23, CK1 phosphorylation site; 24, GSK3 phosphorylation recognition site. (D2.3). *Motif for proteinase cleavage (dark blue colored)*. 31, canonical motif for proteinase cleavage site. (D2.4). *Motif for protein-protein binding (light blue colored)*. 41, Docking motif in calcineurin; 42, USP7 MATH domain binding motif; 43, USP7 CTD domain binding motif; 44, WW domain interaction motif. (D2.5). *Motif for nucleocytoplasmic shuttling (brown colored)*. NLS, nuclear localization signals; NES, nuclear export signals. Most of the predicted motifs for two pairs are identical, except a few motifs which are located at the beginning of transcript (exon 1 for A group, exon 1A for B group).

We have previously detected the cellular protein expression of CCM2 isoforms with multiple bands in Western blots (data not shown), which could be addressed with different interpretations. One major reason (or artifact) causing multiple protein bands is due to various post-translational modifications (PTMs) as indicated by our bioinformatics data (Fig. 3A). 293 T (derived from Human Embryonic Kidney 293) cells were treated with most available PTM inhibitors with no observed band shifts or intensity changes eliminating this possibility (Fig. 3B). Further, silencing CCM2 RNAs in 293 T cells significantly decreased intensity of all bands (Fig. 3C), which eliminates another potential source of artifact caused by non-specificity of the antibody. Our current data validate the belief that multiple CCM2 isoform proteins are expressed in cells although expression levels of each isoform are quite different (Fig. 3B,C).

#### Newly identified exons and isoforms suggest a novel domain in CCM2 protein

We have previously reported that despite lacking either a nuclear localization signal (NLS) or a nuclear export signal (NES) in its sequence, the original CCM2-100 isoform (canonical CCM2 protein) predominantly exists in the cytoplasm in HeLa cells at steady state, but shuttles in between the nucleus and cytoplasm^7^. Since all identified CCM2 isoforms share similar coding sequences with CCM2-100 isoform and contain neither a nuclear localization signal nor a nuclear export signal, most CCM2 isoforms from both groups were predicted to behave similarly as CCM2-100 isoform does within mammalian cells, which contradicts our current data (Fig. 2B, Table 3, Suppl. Fig. 2). We reinvestigated CCM2 protein domains and motifs with multiple bioinformatics tools. By comparing the two longest isoforms in each group (A, B), a PTB domain at N-terminus and an HH domain at C-terminus were found in all paired-isoforms as predicted. However, surprisingly, we not only identified the existence of both NES and NLS in A group (only NES detected in B group), we also detected a novel unknown domain in the middle of CCM2 protein that contains newly defined exon 6A (Fig. 3D), which is in line with our earlier description of an unknown domain (DUF1722) (Suppl. Table 3) predicted from bioinformatics search on both CDD and Pfam databases.

### Newly identified isoform B group is dominant despite A group RNAs being more stable

#### Newly identified isoform B group is the dominant group of endogenously expressed isoforms

**Figure 4:**
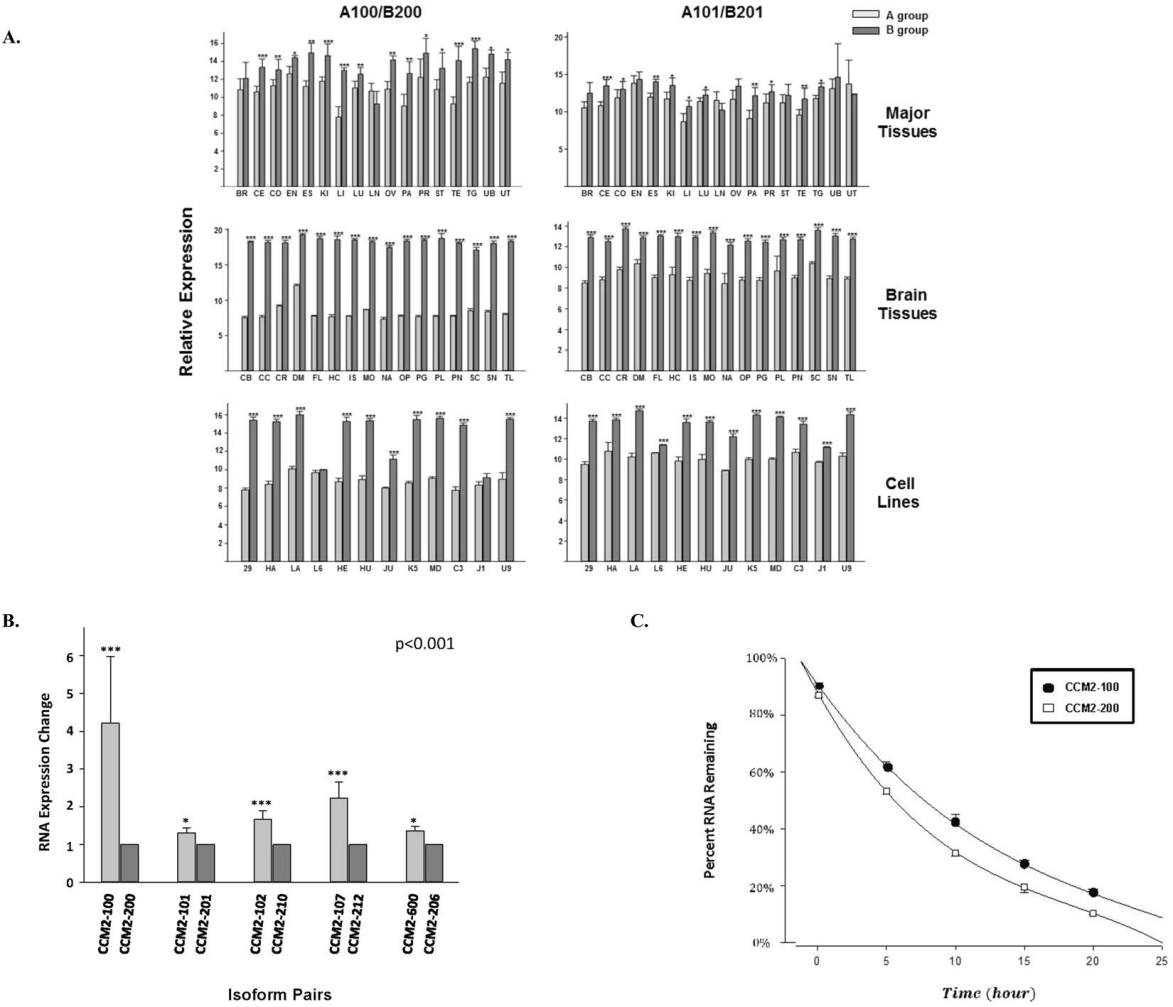
The relative expression level and cellular stability between A and B group isoforms. (**A**) Comparison of endogenous expression levels between CCM2 isoform pairs among various tissues. The relative mRNA expression levels of paired-CCM2 isoforms (2^−∆CT^) were presented with bar plots, in which light grey bars represent A group isoforms, dark grey bars represent their respective counterparts, B group isoforms. For experimental design, the left three panels represent expression levels between CCM2 isoform pairs with primer set, CCM2-A100 and CCM2-B200; the right three panels for CCM2 isoform pairs with primer set, CCM2-A101 and CCM2-B201. For tissue location and cell lines, upper two panels represent the expression levels of paired-CCM2 isoforms among major tissues (see Fig. 2A), middle two panels for various brain tissues, and lower two panels for multiple cell lines (see Suppl. Fig. 1) Middle and lower panels are further described in supplemental Fig. 1. One-way ANOVA was also performed for the comparison between A and B groups of isoforms among different tissues and cells; it was found there is a very significant difference (P < 0.001). The detailed information for isoform-qPCR primer sets is listed in Suppl. Table 2. (**B**) The changes in the expression levels between ectopic expressed A and B group isoform pairs. The expression levels were measured by allele-specific qPCR (V5-tag), then normalized first by internal expression control (Neo) and followed by mean values for B group isoforms, presented as fold changes. (**C**) Comparison of RNA decay rates between two ectopic expressed CCM2-100 (**A**) and CCM2-200 (**B**), isoform pairs from groups A and B respectively, measured with allele-specific qPCR primers (Suppl. Table 2) at five different time points (after 5, 10, 15, 20, and 25 hours). The solid circle presents A Group isoform (CCM2-100), while hollow square presents Group B isoform (CCM2-200). One-way ANOVA was also performed for the comparison between A and B groups of isoforms and found there is a very significant difference for the expression levels between A and B groups of isoforms (P < 0.001). ***, **, and *above bar indicate P =< 0.001, 0.01, and 0.05 respectively for paired *t*-test. For major tissue abbrevia*t*ions refer to Fig. 2; for brain tissue and cell line abbreviations please refer to Suppl. Fig. 1.

To delineate the intracellular relationship between two major groups of CCM2 isoforms, which are expressed ubiquitously in various tissues and cell lines (Fig. 2A, Suppl. Fig. 1), we examined the difference of relative expression profiling between A and B group by using qPCR with two sets of primers (A100/B200 and A101/B201, Suppl. Table 2). On careful evaluation of the data, it was found that the relative mRNA expression level was almost always higher for the newly identified mammalian-specific B group isoforms (Fig. 4A). Further, the expression level differences were even more significant in the more homogeneous tissue samples (brain) and cell lines (middle panel and lower panel, Fig. 4A), further validating this observation.

#### Ectopic expression levels of isoform A group is higher than that of isoform B group

To investigate the molecular mechanism of the consistently higher expression of CCM2 B group isoforms in mammalian cells, we performed qFCM assay with V5 tag-specific antibodies to precisely measure the expressed protein levels at steady state for 5 ectopic expressed isoform pairs between A/B groups (CCM2-100/CCM2-200, CCM2-101/CCM2-201, CCM2-102/CCM2-210, CCM2-107/CCM2-212, and CCM2-600/CCM2-206). In contrast to our endogenous data, flow cytometry results showed that the protein expression level of CCM2 A group isoforms was always higher than that of CCM2 B group isoforms within each pair and the overall difference was found to be statistically significant (p < 0.01) (Table 4), demonstrating the relative higher expression of A group protein isoforms at translational level. Using tag-specific (V5) and vector control (Neomycin) primer sets (Suppl. Table 2), we further performed allele-specific qPCR to examine the RNA abundance within the same 5 isoform-pairs and found very significant differences within each pair, with the overall difference also being highly statistically significant (p < 0.001) (Fig. 4B). Our qPCR data further supported our qFCM results, indicating the notion that the consistently higher protein level of A group CCM2 isoforms (Table 4) is rooted from their significantly higher expression at the transcriptional level (Fig. 4B).

**Table 4:** Differential protein expression levels between A and B groups of CCM2 isoforms.

Isoforms	Relative Expression Level			
	V5	Neo	GMFI Index	Group A/B **
CCM2-100 (A)	44.9	1.7	26.44	2.39
CCM2-200 (B)	4.97	0.45	11.04	
CCM2-101 (A)	22.5	1.1	20.46	2.40
CCM2-201 (B)	7.09	0.83	8.54	
CCM2-102 (A)	40.5	1.48	27.33	2.92
CCM2-210 (B)	8.33	0.89	9.36	
CCM2-107 (A)	64.1	2.73	23.48	2.13
CCM2-212 (B)	31	2.81	11.01	
CCM2-600 (A)	14.7	0.74	19.86	1.56
CCM2-206 (B)	8.27	0.65	12.72	

The geometric median fluorescence intensity (GMFI) is used to account for the log-normal flow data for each isoform and normalized by Neomycin (Neo) signals as GMFI index. The fold changes of expressed proteins between two groups were presented as ratio (A/B Group). One-way ANOVA was performed among all isoform pairs from A and B groups and a significant difference is defined (P < 0.01, **).

#### Isoform A group RNAs are more stable than isoform B group RNAs

We next measured the cellular stabilities of a representative set of ectopic expressed paired CCM2-100 (A group) and CCM2-200 (B group) isoforms at the transcriptional level. Our data demonstrated that the mRNA half-life of CCM2-100 was approximately 1.3 times longer than the CCM2-200 (Fig. 4C). This finding further corroborates with our allele-specific qPCR data that the consistently higher relative expressional levels of A group CCM2 isoforms at the transcriptional level is due to the longer half-life of their mRNAs, compared to B group CCM2 isoforms. (Fig. 4C).

### CCM2 contains a novel second PTB domain

#### CCM2 PTB-less isoforms are able to bind to NPXY motifs

**Figure 5:**
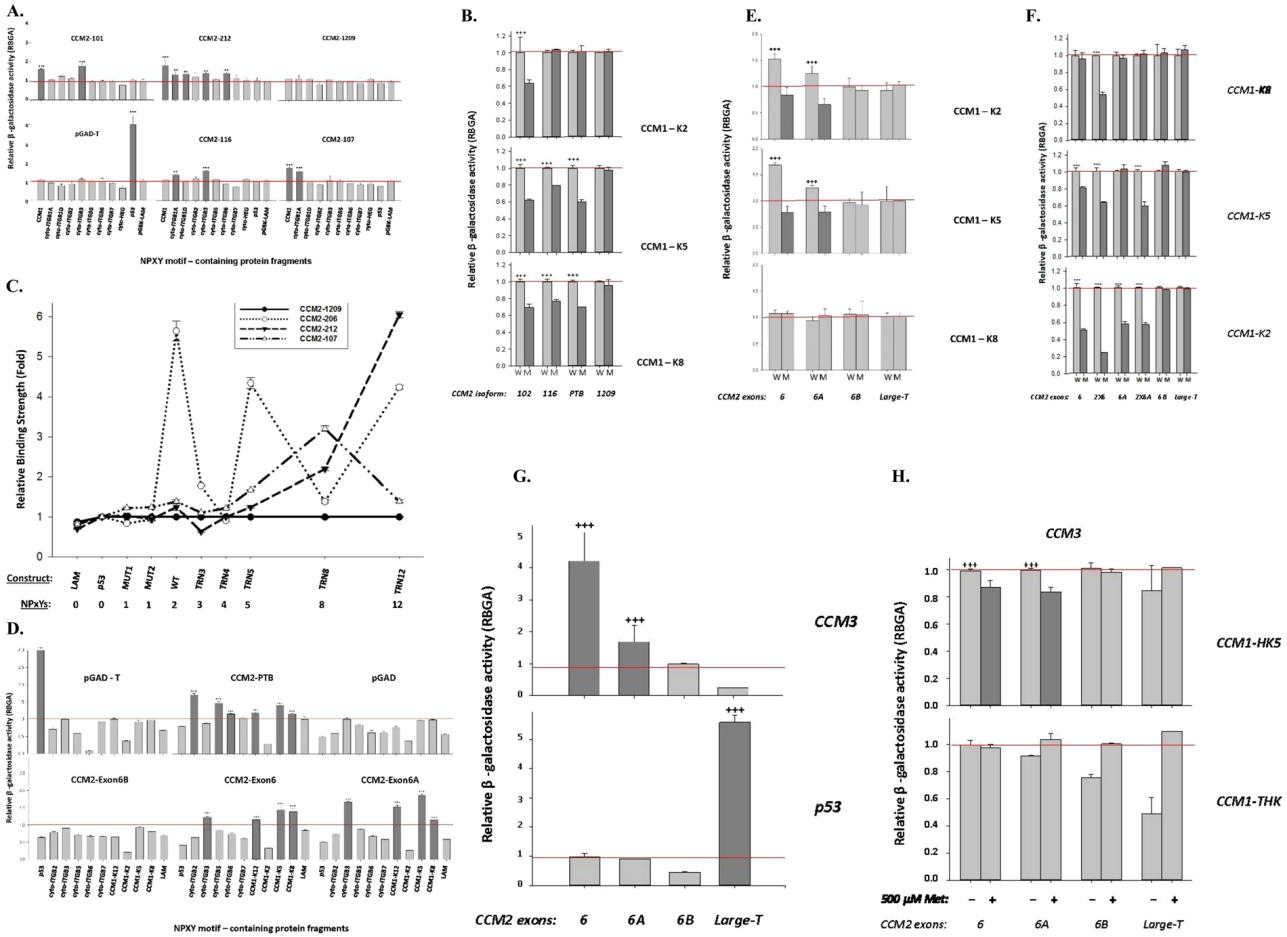
Molecular interactions defined with yeast two-hybrid system. (**A**) Interactions between various NPXY-motif containing protein fragments and CCM2 PTB-less isoforms (CCM2-116, CCM2-107, and CCM2-212). CCM2-101 serves as positive control, while CCM2-1209 as negative control. pGAD-T with p53 is a system control. (**B**) Interactions between wild type (W) and mutated (M) three NPXY motifs of CCM1 (K2, K5, and K8) and a CCM2 PTB-less isoform (CCM2-116). CCM2-102 and full-length CCM2 PTB domain serves as positive control, while CCM2-1209 as negative control. (**C**) Interactions between protein fragments containing various number of NPXY-motifs and CCM2 PTB-less isoforms (CCM2-107, and CCM2-212). CCM2-206 serves as positive control, while CCM2-1209 as negative control. (**D**) Interactions between various NPXY-motif containing protein fragments and CCM2 exons (6, 6A, and 6B). CCM2-PTB serves as positive control, while pGAD as negative control. pGAD-T with p53 is a system control. (**E**) Interactions between wild type (W) and mutated (M) three NPXY motifs of CCM1 (K2, K5, and K8) and CCM2 exons (6, 6A, and 6B). Large-T serves as system control. (**F**) Interactions between wild type (W) and mutated (M) three NPXY motifs of CCM1 (K2, K5, and K8) and CCM2 exons (6, 6A, and 6B) and duplicate forms (2 × 6 and 2 × 6A). Large-T serves as system control. (**G**) Interactions between CCM3 protein and CCM2 exons (6, 6A, and 6B). Large-T with p53 serves as system control. (**H**) Competition assays between CCM3 protein with either CCM1-HK5 (containing 1^st^ NPXY motif) (upper panel) or CCM1-THK (containing 2^nd^ and 3^rd^ NPXY motif) (lower panel) binding to CCM2 exons (6, 6A, and 6B). β -galactosidase activity of each transformant was measured, normalized, and converted to relative β -galactosidase activity (RBGA). The normalized data were represented with means and standard deviations (M ± SD) generated from at least three independent assays (n = 3). RBGA^+++^, ^++^: significantly higher than that observed in any negative controls (P < 0.001, 0.01 respectively). K2, K5, and K8 are fragments containing the first, second, and third NPXY motif in CCM1 respectively. Cyto-ITGB represents cytoplasmic tails of β integrins (usually containing two NPXY motifs).

Among all CCM2 isoforms with PTB domain that we defined, three isoforms (CCM2-107, 116, and 212) do not have apparent classic PTB domain, termed PTB-less (missing exons 3, 4, and 5 coding for PTB domain) (Suppl. Table 4). We used these three isoforms to screen many proteins that contain NPXY motif through yeast-two hybrid system (Y2H). Intriguingly, these isoforms were found to bind efficiently to NPXY motifs (Fig. 5A); mutagenesis analysis further confirmed their binding to NPXY motifs in CCM1 protein (Fig. 5B). Further, constructs with increasing number of NPXY motifs (0–12) showed an overall trend of enhancing binding affinity in the PTB-less isoforms, although this trend is not linear, suggesting there is some specificity to the binding possibly associated with structure conformation (Fig. 5C). In sum, these experiments imply the existence of the second functional PTB domain in the middle of CCM2 protein.

#### The second PTB domain of CCM2 is an atypical PTB domain with novel structure

**Figure 6:**
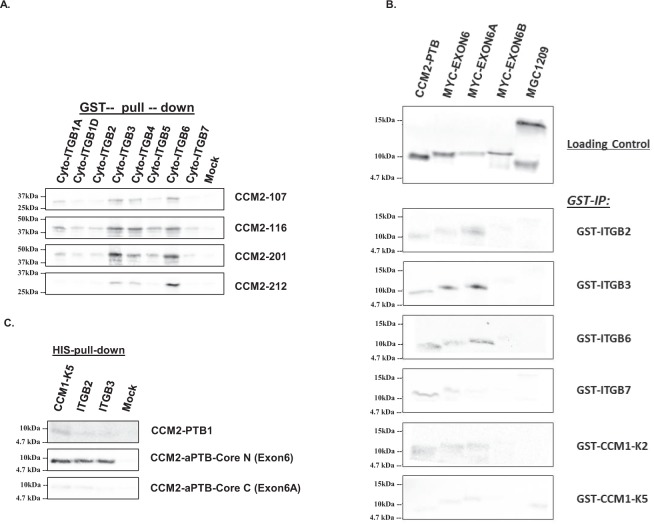
Molecular interactions defined by co-immunoprecipitation (CO-IP). (**A**) Various NPXY-motif containing protein fragments pulling down PTB-less CCM2 isoforms (CCM2-107, CCM2-116, and CCM2-212). CCM2-201 serves as positive control. Interactions between cytoplasmic tails of β-Integrins and CCM2 isoforms were confirmed by IP with GST beads pull-down. (**B**) Various NPXY-motif containing protein fragments pulled down CCM2 exons (6, 6A, and 6B) with GST beads. Full-length CCM2 PTB domain serves as positive control, while CCM2-1209 as negative control. (**C**) Various NPXY-motif containing protein fragments pulling down CCM2 exons (6, 6A, and 6B) with HIS beads. NPXY motifs containing protein fragments are cytoplasmic tails of β-Integrins and CCM1 or PTB cores. CCM2-1209 (CCM2 C-terminal fragment, residues 303-444, no PTB domain), CCM1 fragments, K2 (1^st^ NPXY motif), K5 (2^nd^ NPXY motif), and mock control. Mock control is host cell lysate. MagneGST Glutathione Particles (Promega) for GST-tagged bait proteins and dynabeads (Invitrogen) for HIS-tagged bait proteins. All target proteins were labeled with radioactive with S^35^.

Since our genomic and bioinformatics data indicated the second PTB domain is highly likely located within the predicted new domain in the middle of CCM2, encompassing exon 6 and 6A (Fig. 3D, Suppl. Table 3), we next tested the binding ability of NPXY motifs to the constructs containing amino acid sequences around exon 6 up till exon 7, which include exon 6, exon 6A, and exon 6B with CCM2 full-length PTB domain as a positive control and CCM2-1209 isoform containing solely HH domain (encompassing C terminal exon 7, 8, 9, and 10) as a negative control through yeast-two hybrid system. Both exons 6 and 6A but not exon 6B were found to bind to NPXY motifs (Fig. 5D), and mutated NPXY motifs in CCM1 protein lost their binding capacity to both exons 6 and 6A (Fig. 5E), while constructs with duplicated (2X) exons 6 and 6A, increase their binding capacity to NPXY motif (Fig. 5F). Interestingly, it has been previously reported that CCM3-CCM2 interaction is mediated through LD (Leucine-Aspartate Repeat) motif-like region within exon 6 of CCM2^17^, which is the consensus sequence-determined binding^18,19^. After examining the binding of exons 6, 6A, and 6B to full-length CCM3, it was found that both exons 6 and 6A independently interact with CCM3 protein (Fig. 5G). Further, this interaction between CCM3 and exons 6/6A can be inhibited by CCM1 fragment HK5 (containing the 1^st^ NPXY motif) but not CCM1 fragment THK (containing the 2^nd^ and 3^rd^ NPXY motifs) (Fig. 5H)^2,7,20^, suggesting that the first NPXY motif of CCM1 can compete binding to aPTB domain of CCM2 with CCM3. Together, these results indicate that exons 6 and 6A can independently interact with NPXY motif. The interactions between NPXY motifs were further confirmed in western blots both with PTB-less isoforms (Fig. 6A) and with exons 6 and 6A (Fig. 6B,C), validating this newly defined interaction observed in Fig. 5F.

In sum, we found that both protein fragments coded by exon 6 and exon 6A can independently interact with various NPXY motifs confirmed by Y2H (Fig. 5), Co-IP assays (Fig. 6), and characterized with BLI and ITC results (Table 5). Protein fragments coded by exon 6 and exon 6A were named as aPTB-core-N and aPTB-core-C respectively (Fig. 6C).

**Table 5:** Kinetic analysis of the interactions between NPXY motif of CCM1 and two cores of novel aPTB domain.

*Method*	BLI	ITC	
*Affinity*	*KD (M)*	*KD (M)*	
*Ligand*	CCM1-K8	CCM1-K8	CCM1-K2
CCM2-EX6	4.06 × 10^−6^	1.99 × 10^−6^	6.45 × 10^−6^
CCM2-Ex6A	2.97 × 10^−5^	NA	6.02 × 10^−5^
CCM2-Ex6B	NA	NA	NA

Affinity data obtained using two different platforms (BLI and ITC) were presented. Binding affinity is presented with the equilibrium dissociation constant (K_D_). NA: not available due to undetectable binding.

#### The binding of atypical PTB domain of CCM2 to CCM1 and CCM3 is structurally determined

**Figure 7:**
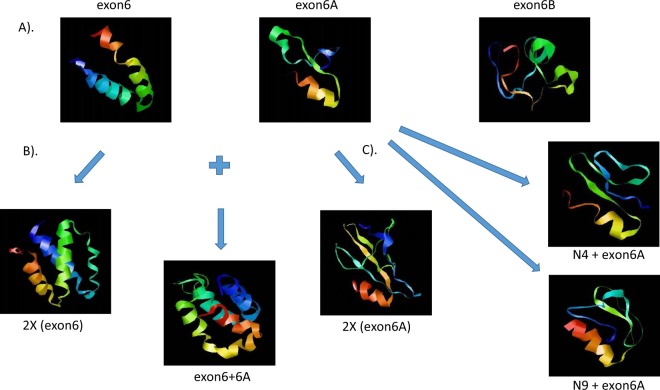
Structures of the novel atypical PTB domain. (**A**) Ribbon representations of exon 6, exon 6A and exon 6B. Both exon 6 and exon 6A share a structural similarity with a C-terminal α helix followed by a turn, but not exon 6B. (**B**) Predicted ribbon presentation of CCM2 exon homo-and hetero-dimers. Homodimer of exon 6 (2X exon6), homodimer of exon 6A (2X exon6A) and heterodimer of exon 6 and 6A (canonical aPTB domain, exon6 + 6A) were simulated. Surprisingly, homodimer of exon 6 (2X exon6) was found to assemble to a naturally occurring heterodimer between exon 6 and 6A (exon6 + 6A), a dual horseshoe or simple duplication of exon 6, raising the doubt of the accuracy of predicted structure of exon 6A. (**C**) To determine accuracy of predicted structure of exon 6/6A, 4 or 9 amino acids from exon 6 were added to C-terminus of exon6A (N4 + exon6A and N9 + exon6A respectively). N4 + exon6A is equal to exon 6 in size (45 aa), while N9 + exon6A is larger than exon 6 in size (50 aa) and contains > 20% of amino acid sequence from exon 6, suggesting that neither size nor sequencing from exon 6 influences conformation of exon 6A. Red color: c-terminus and blue color: N-terminus.

Our predicted tertiary structure data show that exon 6 (aPTB-core-N) is a novel structure that has not been reported before, which is shaped as a horseshoe composed of two α-helices (Fig. 7A). In contrast, exon 6B fragment does not bind to NPXY motif, due to absence of this structure. Interestingly, the initial predicted tertiary structure of exon 6A fragment seems to be a structurally immature analog of exon 6, with only one crude C-terminal α-helix, a turn and flexible N-terminus. However, in the presence of its partner exon 6 sequence, it matures to become an exon 6-like structure, a horseshoe composed with two α-helices. Both exon 6 and exon 6A linked as two horseshoes form a serine-rich four alpha helix bundle (hairpin), structurally similar to that of the homodimer of exon 6 (CCM2-exon6x2) (Fig. 7B). Interestingly, this novel four-helix bundle (45 aa/horseshoe) is structurally similar to but smaller in size than previously reported serine-rich four-helix bundle focal adhesion adaptor protein, p130-CAS (55–60 aa/ horseshoe)^21,22^. To determine whether the sequence or the length of amino acid chain at N-terminal exon6A could cause this structure to transition to a “mature” exon6 horseshoe structure, 4 (same length of exon 6) and 9 (5aa longer than exon 6) amino acids from C-terminus of exon6 were added to the N-terminus of exon6A respectively; however, the tertiary structure did not show significant shift to assemble the structure of a “mature” exon6, which suggests there are some issues hindering the correct prediction of exon6A as horseshoe composed with two α-helices, which we believe is the case, from bioinformatics (Fig. 7C).

Sequence comparison showed that there is a possible LD motif-like region present in exon 6, but no such homolog can be found in exon 6A (Suppl Table 4). Therefore, the interaction between exon 6A and CCM3 can’t be mediated through LD-motif like sequence (Fig. 5G,H and Suppl Table 4). The possibility that exon 6 and exon 6A interact with CCM3 through different mechanisms seems minimum, therefore, due to the similarity in tertiary structure between exon 6 and exon 6A, and the competitive nature of both cores with the 1^st^ NPXY motif of CCM1, we conclude that the binding between CCM2 and CCM3 is structurally determinant rather than sequence determinant (Figs 5, 7).

By analyzing these data, we conclude that the newly defined atypical PTB domain (aPTB) is composed of exon 6 and exon 6A, each approximately 45 amino acids long, and can independently bind to either CCM1 or CCM3 proteins. In fact, this binding potential can be augmented when both form homo- or hetero- dimers folding into structurally identical four-alpha helix bundle structure (Figs 5F, 7B). The homodimer of exon 6 has the better tendency than exon 6 alone to bind to the NPXY motif of CCM1, suggesting that the binding between CCM2 aPTB domain and the 1^st^ NPXY motif of CCM1 is mediated through tertiary structure, similar to the binding between CCM2 PTB and the 2^nd^ and 3^rd^ NXPY motif of CCM1-canonical PTB-NPXY binding. Since, the full aPTB domain present in CCM2 isoforms is a four alpha helix bundle composed of exon 6 and exon 6A, it can be reasonably assumed that the minimum requirement for binding between aPTB of CCM2 and NPXY motif is the existence of C-terminal alpha helix and turn (highly possible including the 2 alpha helix as the horseshoe for its basic functional unit) of both cores of aPTB domain of CCM2.

## Discussion

### This current report may help define more CCM mutations

Upon comparing linkage results^23^ and mutational screening data^24^ of known *CCM* genetic loci (*CCM 1–3*), there is a noticeable discrepancy in the relative incidence of CCM mutations among CCM patients, raising the possibility of the existence of an additional *CCM4* locus^25^. However, endeavors to find the new locus have thus far failed. Based on our previous RNA and protein data on CCM2, we hypothesized that the additional exons might exist within *CCM2* genomic structure, contributing to this discrepancy. In this report, we have defined a total of 29 exons, including 8 new exons and 11 alternatively spliced exons derived from existing exons and 5 novel promotors in *CCM2* gene. Using our designed primers (Suppl. Table 1), we screened a small group of previously defined CCM2 patients. Ironically, we still could not identify novel CCM mutations in these newly identified exons, which could be due to our small cohort of CCM patients. As per our past experience, the lack of resources could hinder our efforts to discover novel *CCM* mutations. However, these newly identified 22 novel functional CCM2 isoforms have proved the biological importance and relevance of transcriptional alternative splicing of *CCM2* gene and are further validated by our expression data at both transcriptional (Fig. 2A, Suppl. Fig. 1A,B) and translational (Fig. 3A,B) levels. Although almost all reported mutations in *CCMs* are nonsense mutations^6,26–29^, there are reports for abnormal splicing mutations^30,31^, raising the possibility of more mutations existing in the newly identified alternative splicing sites. Therefore, the information and evidence generated in this study will certainly facilitate the rapid identification of the remaining undefined mutations at *CCM2* locus in the near future.

### Alternative splicing of CCM2 may be a key modulator of CSC during angiogenesis

Alternative splicing of a transcript can result in multiple protein isoforms with divergent functions from original “full-length” protein and more than 95% of human genes have been found to utilize this mechanism to increase the complexity of gene expression by producing protein isoforms with different, even opposing functions^32^. Alternative splicing has emerged as one of the key modulators of angiogenesis^33^ and current evidence suggests that different isoforms arise from alternative splicing of certain angiogenic molecules such as vascular endothelial growth factor (VEGF)^34^, VEGF receptor1, flt1^35^, and integrin receptor: fibronectin (FN)^36^. Alternatively spliced isoforms of VEGF can be either proangiogenic or antiangiogenic^34,37–39^, emphasizing the biological significance of this RNA processing mechanism. It has been demonstrated that CCM2 works with other CCM proteins to regulate β1-integrin-mediated cellular events through the CSC complex^7,8,12^. Although we found that both CCM1 and CCM3 are less polymorphic regarding alternative splicing events in our hands, an alternative spliced CCM1 isoform lacking the 15th coding exon, Krit1B, has been reported to be expressed at relatively high levels in mouse tissues and cell lines, but much less expression detected in humans^40^; interestingly, an “in-frame deletion” found in a large CCM cohort mutation screening, which results in the lack of entire exon 18 of *CCM1* gene, was found to have dramatically decreased expression level as well^41^. For isoform Krit1B, the splicing out of the 15th exon coding region occurs within F3 lobe of the FERM domain, raising the possibility of disrupted intramolecular interaction and folding^42^. This in-frame deletion region (residues 675–714) is actually next to previously identified nuclear export sequence (residues 551–562)^7,8^ and a nuclear localization signal (residues 569–572)^43^ with potential impact on both functions. Therefore, it is understandable that the rare CCM1 isoform, Krit1B, behaves differently from the dominant cellular CCM1 isoform, Krit1A, in the cellular compartmentation. Likewise, the differential nuclear localization of some CCM2 isoforms in endothelial cells in this experiment can be explained.

Ccm2-like (Ccm2L), a recently described novel gene with a high homology to Ccm2 in zebrafish, was identified^44^. Like Ccm2, Ccm2L has multiple alternatively spliced isoforms and works within the CSC complex (Ccm1 and Ccm3) to interact with the Mekk3/Mek5 complex to regulate Mekk3 activity^45^. However, no studies have been performed on CCM2 isoforms till date. More than half of human genes have alternative promoters with multiple start-codon exons^46,47^, but only a few of them display sequence similarity within their first coding exons, giving a variety of closely related N-terminal protein variants^46,48^, and CCM2 certainly belongs to this small group.

#### Differential expression between two major isoform groups regulated at transcriptional level

This project is highly successful in deciphering the two major types of CCM2 isoforms, which differentiated themselves by alternative promoters and alternative start codon exons, categorized into A and B group. It is very interesting to observe that despite the subtle differences at their 5′ end sequences (30 bp for exon1, 93 bp for exon1A) the transcripts of isoform pairs from two groups behave quite differently in their cellular abundance at steady state. The *in-vivo* qPCR data demonstrated that the transcripts of mammalian-specific B group isoforms are more abundant than that of vertebrate-specific A group isoforms in almost all mammalian cells and tissues screened, hinting the preferred higher endogenous expression levels for B group isoforms at the transcriptional level in mammalian cells, at steady state. However, protein expression data were totally incongruent to the endogenous expression data generated from tissues and cell screening at the transcriptional level. There are a few possible molecular mechanisms resulting in this discrepancy such as the stabilities of isoforms at either post-translational and/or post-transcriptional levels. The stability of mRNA transcripts was investigated, and surprisingly demonstrated that the vertebrate-specific A group isoforms are more abundant (Fig. 4B). We further measured the RNA decay rate of one set of isoform pair representing both groups and found that the mRNA half-life of vertebrate-specific A group isoform is indeed longer than the mammalian-specific B group isoform (Fig. 4C). Our results are in line with a recent finding that differential usage of alternative first exons increases the capacity for transcriptional control of alternative spliced isoforms^48^, however our data also raises a very interesting suggestion that B group isoforms must have a stronger promoter than A group isoforms to ensure their dominant expression in most tissues and cells.

There have been many reports indicating that both alternative promoter usage^47,49–51^ and 5′ untranslated region (5′ UTR)^50,51^ regulate the alternative splicing event both quantitatively (amount of isoform) and qualitatively (the species of isoform), making things even more complicated. Eukaryotic transcription is tightly controlled by various cis-DNA regulatory elements, such as enhancers, boundary elements, insulators, and silencers. Considering that the exon1A is more than 27 kb downstream of the exon1, it is unlikely for any overlapped promoter regions between two isoform groups to exist. Our findings that mammalian-specific B group isoforms are less stable but more abundant than the original vertebrate-specific A group isoforms at steady state are quite intriguing and the stronger transcriptional machinery, regulated by a specific set of regulatory elements (P1-P5), for the B group isoforms is the most reasonable explanation for the contradictory phenomenon. Since cell-type specific transcription is regulated by specific combinations of transcription factors on the specific DNA region for the connectivity of gene transcription regulatory networks at the quantitative level, we had acquired BAC clones which contain several kb upstream of either exon1 (A group) or exon1A (B group) for future discovery and characterization of both cis- (promoter, 5′UTR, its proximal region, its enhancers, boundary, or insulator elements) and trans- (transcription factors) elements associated with their transcriptional regulation.

#### Differential subcellular compartmentation of the isoforms indicates their distinct cellular roles

Scaffold proteins are critical in regulating the specificity of signaling responses through mediating the assembly of distinct multimolecular signaling complexes, or signalosomes that mediate appropriate responses. CCM2 has been defined as a scaffold protein. CCM2 isoforms which contain a PTB domain are more likely to interact with CCM1 and may use it as a cellular shuttle as well between the nucleus and cytoplasm. However, new nuclear localization and nuclear export signals have been identified in CCM2 in this study, raising the doubt for the previous hypothesis. Interestingly, even PTB-less isoforms (CCM2-107, CCM2-116, and CCM2-212) frequently distribute evenly in both the nucleus and cytoplasm at steady state in several cell lines (mainly in endothelial cells), further challenging the previous hypothesis. There have been reports of differentiated cellular compartmentations among different alternatively spliced isoforms of other PTB-domain containing proteins^52,53^, suggesting that there may be a common mechanism for the intracellular distribution of PTB-domain containing proteins modulated through alternative splicing. These findings underscore the importance of the pleiotropic nature of CCM2 proteins through its molecular strategy of alternatively spliced isoforms, with each isoform being possibly functionally distinct from its siblings.

### CCM2 plays essential role as a cornerstone of CSC

**Figure 8:**
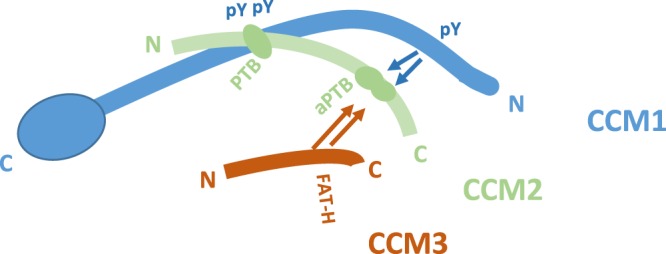
Schematic representation of binding interaction among CCM proteins within CSC complex. Our current data suggests that CCM1 utilizes 2^nd^ and 3^rd^ NPXY motifs (pY) in its center portion to bind to CCM2 classic PTB domain (PTB). The remaining 1^st^ NPXY motif competes with the CCM3 (FAT-H) to bind to the newly defined atypical PTB domain (aPTB) present at the C – terminus of the CCM2, suggesting CCM2 plays a central role in the CSC. CCM1: blue color; CCM2: light green color; CCM3: red color.

In this study, the most important finding is the discovery of previously unknown atypical PTB domain (aPTB) in CCM2, which consists of two cores that can independently bind either CCM3 or CCM1. For our data, the aPTB domain seems to be able to bind to all three NPXY motifs but prefers the 1^st^ motif, probably due to conformation and accessibility issues in the full-length CCM1. Intriguingly, CCM3 and CCM1 can compete for their binding to aPTB domain of CCM2, making us propose a model to demonstrate their relationship within the CSC complex (Fig. 8). In this model, CCM1 protein can use its 2^nd^ and 3^rd^ NPXY motif in the middle portion of the molecule to bind to the longer CCM2 isoforms through its N-terminal PTB domain and use its 1^st^ NPXY motif at N-terminus to bind to aPTB domain of CCM2, which also binds to CCM3. We have proved the competitiveness of the 1^st^ NPXY motif of CCM1 and CCM3 binding to aPTB domain of CCM2, but in an overexpressed system. Since there are two independent cores in aPTB, the interaction between CCM1 and CCM3 with aPTB, whether cooperative or even independent in the physiological condition, needs to be further explored.

In summary, our data suggest that CCM2 can play pleiotropic cellular roles by enhancing the variation of its coding sequences and domains through alternative splicing processes in various cells and tissues. We illustrated that alternatively spliced CCM2 isoforms have isoform-specific cellular expression and subcellular localization patterns among different cells and tissues. Our data validate our previous belief that CSC can mediate signaling activities of multiple pathways through its multiple molecular forms. In conclusion, we proposed that the differential cellular expression and subcellular localization patterns are partially due to the difference of transcriptional regulation among different isoforms. Various isoforms also diversify its cellular localization and dynamics, creating multiple layers of representation of this single protein. Combination of these cellular events will certainly increase the complexity of CSC mediated events in angiogenesis. This project provides new insights into CSC-mediated signaling pathways and angiogenesis, which may revolutionize the current concepts of vascular malformations and molecular mechanism of angiogenesis, leading to new therapeutic strategies.

## Materials and Methods

### Isolation of novel exons, open reading frame, isoforms, and conserved domains in *CCM2* gene

Multi-tissue reference RNAs pool from four different suppliers (Super Array, BiotaQ, Biochain, NTomics) were used to amplify CCM2 cDNA fragments with SuperScript® III reverse transcriptase (Invitrogen). Rapid amplification of cDNA end (RACE) was applied for using either human brain Marathon-Ready cDNA or SMART RACE kit reagents (BD Clontech) to define full-length cDNA. A second round of amplification was performed to enhance the specificity and yield using nested primers (Suppl. Table 1).

The RACE and RT-PCR resulting products were sequenced and further analyzed with Vector NTI (Invitrogen) and CLC genomic workbench (Qiagen) to define the potential novel fragments and open reading frames (ORFs) as described before^43^. After the start-codon exons of isoforms were defined, all full–length CCM2 isoforms were amplified from the cDNA pool with the proper primers and Platinum Pfx50 DNA Polymerase (Invitrogen) (Suppl. Table 1). Potential conserved domains (CD) on the protein were searched on both CDD and Pfam databases in NCBI CD search site (http://www.ncbi.nlm.nih.gov/Structure/cdd/wrpsb.cgi).

### Real time quantitative PCR analysis (qPCR)

Allele-specific real-time quantitative PCR (qPCR) assays were designed using boundary-spanning primer sets (Suppl. Table 2) and applied to quantify the RNA levels of the endogenously expressed CCM2 isoforms using Power SYBR Green Master Mix with ViiA 7 Real-Time PCR System (Applied Biosystems). TissueScan™ Real-Time PCR panels (HMRT100, 103, CSRT502) with Human ß-actin control primer set (Origene, MD) were used to determine the endogenous expression levels of various CCM2 isoforms in different tissues. qPCR plates with human brain tissues and cell lines were prepared using an epMotion 5075 automated liquid handling systems (Eppendorf) and were performed in triplicates (n = 3). V5-tag and Neomycin allele-specific qPCR primers (Suppl. Table 2) were used to perform allele-specific qPCR to quantify the ectopic expression levels of each isoform-pair. The qPCR data were analyzed as described before^20^.

### Plasmid construction, subcellular localization, western blots, and cellular dynamics

CCM2 isoforms were inserted into corresponding gene-expression vectors as described previously^7,16,20,43^. The efficient expression for each CCM2 isoform in both transcriptional and translational levels was first confirmed through both qPCR and Western blots and subsequently enabling further experiments, such as transient gene expression associated with immune-fluorescence (IF) analysis, quantitative flow cytometry (qFCM), and RNA decays assays. Anti-CCM2 (Sigma), β-Actin, and α-Actinin (Santa Cruz) antibodies with anti-GST (Promega) and HIS-tag (Invitrogen) beads were used in Western Blots.

Multiple primary endothelial (HUVEC, HMVEC, hCMEC/D3) cell lines were cultured in six well plates and transfected with different CCM2 isoform-V5 constructs to determine their subcellular localization and expression level as described^2,7,8,14,54,55^. Transfected cells were then fixed, and subcellular localization of each isoform was determined with IF analysis using NIKON TiE imaging system. Cyclin B, known to accumulate in the nucleus after leptomycin treatment, served as a positive control during IF screening.

For qFCM assay, several isoform-pairwise constructs (100/200, 101/201, 102/210, 107/212, and 600/206) with nearly identical coding sequence differing only in the start-codon exon, exon 1 (isoform A group) or exon1A (isoform B group) at 5′ terminus, were used to study the protein expression pattern of these two major groups of CCM2 isoforms. The ectopically expressed proteins from each isoform pairwise construct were measured separately in the same batch of 293 T cells with triplicate repeats, with standard transfection regime. Twenty-four hours after transfection, cells were detached with 0.05% Trypsin-EDTA solution (Sigma-Aldrich) and counted in a hemocytometer (American Optical). The cells were fixed in 4% PFA for 20 min at room temperature and then permeabilized by 90% cold methanol on ice for 30 min. The cell concentration was adjusted to 1 × 10^6^cells/mL by blocking buffer and was incubated at room temperature for 1 hour. The cells were further divided in two parts: one was stained with 2 μg/mL sample of anti-V5-FITC mouse monoclonal IgG2a (Invitrogen) and the other one with anti-neomycin phosphotransferase II Rabbit polyclonal IgG (Millipore) and Alexa Fluor 647 Goat Anti-Rabbit IgG (H + L) (Invitrogen). Normal Rabbit IgG (Millipore) followed by Alexa Fluor 647 Goat Anti-Rabbit IgG (H + L) was used as the background control. The expression level of neomycin phosphotransferase II was used as an internal control for transfection efficiency. The cells were washed 3 times with ice cold PBS at 500 g/5 min after each step in cell staining. The cells were then resuspended in 0.5 mL PBS and measured by Gallios^TM^ flow cytometer (Beckman Coulter). The flow cytometry data were analyzed by Kaluza® Analysis Software (Beckman Coulter).

For RNA decay assay, 293 T cells (1 × 10^6^cells/mL) were cultured and transiently transfected with isoform-pairwise CCM2-100/200 constructs as previously described. After 24 hours, 5 μM Actinomycin D (Life Technologies) was added and cell concentration was adjusted to 2 × 10^6^cells/mL by cell counting with a hemocytometer. Treated cells were collected at five different time points (0 h, 2 h, 6 h, 10 h, 24 h) for RNA extraction and qPCR analysis.

### Yeast two-hybrid analysis, protein preparation, and co-immunoprecipitation

Yeast two-hybrid analysis was performed as previously described^2,7,20^. In initial Co-IP experiments, potential target proteins were expressed with TNT® Quick Coupled Transcription/Translation and *E. coli* S30 Extract Systems (Promega) with S35-labeled methionine (PerkinElmer). The expressions were confirmed by Western blots. *In vitro* pull-down co-immunoprecipitation was performed as previously described^2,7,20^.

### Measurement of molecular interactions and modeling of tertiary structures

Recombinantly expressed proteins were purified with chromatography: GST-tagged fragments from PTB domains were purified with GSTrap HP column (GE), while HIS-tagged NPXY motifs were purified using HiTrap TALON column (GE), followed by size-exclusion column, S100-HR (GE). The binding interaction between fragments from PTB domains and NPXY motifs were quantified by Bio-Layer Interferometry (BLI) and Isothermal Titration Calorimetry (ITC). Subsequent data acquisition and analysis were performed as described before^20^.

The molecular modeling of the tertiary structure of atypical PTB (aPTB) domain was established by Iterative Threading ASSEmbly Refinement (I-TASSER), with RasMol (version 2.7.5) for the structure visualization, as described before^20^.

### Statistical analysis

One-way analysis of variance (ANOVA) was used to detect the differences in the mean values among the treatment groups. All pairwise multiple comparison procedures were analyzed using Tukey t-test to test the difference between each treatment. Plots and charts were constructed and produced by SigmaPlot 12.0 (Systat Software, Inc.).

### Ethical approval

This article does not contain any studies with human participants performed by any of the authors, nor studies with animals performed by any of the authors.

## Supplementary information

Supplemental information

## Data Availability

The datasets generated and/or analyzed during the current study are available from the corresponding author on reasonable request. Some data generated or analyzed during this study have been included in this published article (and its Supplementary Information files).
