# Supplementary material for: Alternatively spliced isoforms reveal a novel type of PTB domain in CCM2 protein
Source: Sci Rep. 2019 Nov 1;9:15808. doi: 10.1038/s41598-019-52386-0 (PMC6825194; doi:10.1038/s41598-019-52386-0)

Supplemental information

Alternatively spliced isoforms reveal a novel type of PTB domain in CCM2 protein

Xiaoting Jiang, Akhil Padarti, Yanchun Qu, Shen Sheng, Johnathan Abou-Fadel, Ahmed Badr, Jun Zhang*

Suppl. Table 1. **Primers for the identification and amplification of various CCM2 isoforms**. The primers are listed in three groups, RACE primers, nested primers, and full-length CCM2 amplification primers. The sequence, direction, and intragenic location of each primer are also listed.

Suppl. Table 2. **Primers for the quantitative PCR (qPCR) for the detection of differentially expressed CCM2 isoforms**. The primer pairs starting exon1 and spanning into other downstream exon (exon 2, 3, etc.) junctions were used for detecting A group isoforms; primer pairs starting exon1A and spanning into other following exon (exon2, 3, etc.) junctions for detecting B group isoforms, respectively. The sequence, qPCR product size, potentially covered isoform(s) and optimized annealing temperature of each primer pair are listed. The primer sets A-100 (spanning exon1-exon2 junction and within exon 2) and B200 (spanning exon1A-exon2 junction and within exon 2) represent a collection of comparable isoforms from A and B group, respectively; while the primer sets A-102 (spanning exon1-exon3 junction and exon4-exon7) and B210 (spanning exon1A-exon3 junction and exon4-exon7) for distinguishing a specific pair of isoforms (CCM2-102, CCM2-210). Allele-specific primer sets for V5 tag and neomycin were used for isoform-specific qPCR.

Suppl. Table 3. **The length and distribution of functional domains along the protein structure of the representative isoforms of CCM2**. Total 9 distinct functional domains were identified by search NCBI CDD database. The phosphotyrosine-binding domain (PTB, or phosphotyrosine interaction - PI domain) is listed first due to its interaction with

NPXY motif of CCM1 and β1 integrin cytoplasmic tail. Besides PTB domain, there are additional 8 functional domains: DUF1722 (unknown function) that is partially overlapped with reported LD motif and global domain predicted in this report (Fig. 3D) in the middle of CCM2; SSL2 (DNA or RNA helicase); N5-glutamine S-adenosyl-L-methionine-dependent methyltransferase (both protein methyltransferase and tRNA (guanine-N7-)-methyltransferase activities with ATPase activity); GATase1-like (glutamine metabolic process); tbpA (transmembrane movement); excinuclease ABC subunit A (DNA mismatch repair process); flagellar hook-associated protein L (cellular ciliary or flagellar motility), and PRK07168 (bifunctional uroporphyrinogen-III methyltransferase and/or uroporphyrinogen-III synthase activity). PTB, DUF1722 and SSL2 domains are most abundant domains among all CCM2 isoforms. aa -- amino acid residues. *CCM2-100 isoform is originally reported as canonical CCM2 protein with ten- coding exons.

Suppl. Table 4**. Identification of essential cores of atypical PTB domain in CCM2**. The sequence of exon 6 along with newly identified adjacent exon 6A and exon 6B of CCM2 are compared. Neither their sequences nor their tertiary structure (Fig) share any similarity to the PTB domain in CCM2, by comparing exons with PTB domain; while exon 6 and exon 6A were defined by their specific interaction with various NPXY motifs in cytoplasmic domains of representative β-integrins and CCM1. The LD-like sequence within exon 6 is underlined/Latinized (consensus: LDxLLxxL, Red colored letter: present identical to LD-like sequence; Green colored letter present conserved amino acids to LD-like sequence), which does not share any notable homologs or similarities to the sequence

of exon 6A of CCM2, but share highly similar tertiary structure (Fig. 7).

Suppl. Fig. 1. **Relative expression profiling of endogenous CCM2 isoforms in brain tissues and cell lines by qPCR**. The expression levels of CCM2 isoforms ($2^{-\Delta CT}$) were presented with bar plots, while β-actin was taken as an inference gene. qPCR primer set information for CCM2 isoforms was provided in Suppl. Table 2. A). For Brain tissues: CB, Cerebellum; CC, Cerebral Cortex; CR, Corpus Callosum; DM, Dura Matter; FL, Frontal Lobe; HC, Hippocampus; IS, Insula; MO, Medulla Oblongata; NA, Nucleus Accumbens; OP, Occipital Pole; PG, Paracentral Gyrus; PL, Parietal Lobe; PN, Pons; SC, Spinal Cord; SN, Substantia Nigra; TL, Temporal Lobe. B). For endothelial cell lines: HU, HUVEC; HA, HAEC; HE, HMVEC. For cancer cell lines: MD, MDA231; K5, K562; L6, HL60; U9, U937; C3, PC-3; J1, RAJ1; JU, JURKAT; 29, 293T; LA, HELA.

Suppl. Fig. 2. **Differentiated cellular compartmentations among different CCM2 isoforms in various cell lines**. Each CCM2 isoform was transiently transfected and images show the subcellular localization of CCM2 isoforms with /without treatment of leptomycin. Cell lines: A. Hela cells, B. HUVEC cells, C. HMVEC cells; and D, hCMEC/D3 cells.

Suppl. Fig. 3. **Original digital images at a lower exposure of Western blots in Figures 3 and 6.** Protein lysates were separated by SDS-PAGE using Criterion Precast gels (Bio-Rad), specifically TGX gels for Fig. 3, 4-15% Tris-HCL gels for Fig. 6A and 16.5% Tris-Tricine gels for Fig. 6B and 6C. In Fig. 6B and 6C, all target proteins were tagged with

epitopes and labeled with radioactive with S^{35}.

Suppl. Table 1

PRIMER	SEQUENCE	DIRECTION	LOCATION
**RACE primers:**			
MGC41F	CTCTGTCACCTTCTCATGGGCTTTCTT	**FORWARD**	**intra-exon:2**
MGC42F	CAGGCTTCTTGCCCTTCTTGCCC	**FORWARD**	**inter-exon:1-2**
MGC-6A-F1	AGCCACGTGACCTCTTCCACAGTCA	**FORWARD**	**intra-exon:6A**
MGC-6A-F2	TTCCTTTGACTGTCAGCTTCCTCT	**FORWARD**	**intra-exon:6A**
MGC-6A-R1	AAATAGGGAGATGACCCTGGA	**REVERSE**	**intra-exon:6A**
MGC-6A-R2	TTTGGGTGGGCTCAATGTGATT	**REVERSE**	**intra-exon:6A**
MGC5'RACE-3	GGGAGATGACCCTGGAGAATTTGGGTG	**REVERSE**	**intra-exon:6A**
MGC5'RACE-1	GCCCAGCGCCTCAATGTCGCTGCTGATG	**REVERSE**	**intra-exon:10**
MGC5'RACE-2	CGAAGTGCTGGCTGTCCTTCTCAGGG	**REVERSE**	**intra-exon:10**
MGC5'RACE-4	GGAGGGATTCAGGTATCCTGGTATGGACG	**REVERSE**	**intra-exon:3**
MGC5'RACE-5	CTCAGCTACTCAGGAGGCTGAGGC	**REVERSE**	**intra-exon:2B**
MGC5'RACE-6	CGAAGGGTGGAAACACGCGACAAGGC	**REVERSE**	**intra-exon:1B**
MGC5'RACE-7	CACACCTGCAGTGCCTTGGGATC	**REVERSE**	**intra-exon:1D**
MGC5'RACE-8	CTAGCTGCATTCTCCATGCAAAACATTGAG	**REVERSE**	**intra-exon:1C**
MGC5'RACE-10	GGAGAGATTCTGGTTCCTCCTCTGCCG	**REVERSE**	**intra-exon:1A**
MGC200-F2	CAGAGGGAGGGCTAACATCTGGCCATA	**FORWARD**	**intra-exon:1A**
MGC5'RACE-11	GCTCAGCAGTCTGTCTGGCTCGACGCG	**REVERSE**	**intra-exon:2**
**Nested primers:**			
MGC1A-R1	TCATTCTCCATGGAATACCCTGTGTG	**REVERSE**	**intra-exon:1A**
MGC1A-R2	TTCCTCCTCTGCCGACAGCTACTATGCAT	**REVERSE**	**intra-exon:1A**
MGC1B-F1	CATGAGTCCTGGAGGCAGGTAGAGGC	**FORWARD**	**intra-exon:1B**
MGC1B-F2	CACCTGGGAATGGAGTTATCTCT	**FORWARD**	**intra-exon:1B**
MGC1B-R1	GTTTGCAGTGCTTGCTCAAAGGCAG	**REVERSE**	**intra-exon:1B**
MGC1B-R2	AGATAACTCCATTCCCAGGTGGGGCTT	**REVERSE**	**intra-exon:1B**
MGC1C-F1	CTTTTTAGCACTTTCTCCAGAGT	**FORWARD**	**intra-exon:1C**
MGC1C-F2	ATGTTTTGCATGGAGAATGCAGC	**FORWARD**	**intra-exon:1C**
MGC1D-F1	CTGTTTCTTGGAGTCCACATCT	**FORWARD**	**intra-exon:1D**
MGC1D-F2	GGATGATCCTCGTTTTGCTGGGAGTA	**FORWARD**	**intra-exon:1D**
MGC1D-R1	TCACACCTGCAGTGCCTTGGGATCT	**REVERSE**	**intra-exon:1D**
MGC1D-R2	CTACTCCCAGCAAAACGAGGATCATCC	**REVERSE**	**intra-exon:1D**
MGC1E-F1	TTTGCCTTGAAGAAGTGAGCTTG	**FORWARD**	**intra-exon:1B**
MGC1E-F2	ATGAGTCCTGGAGGCAGGTAGAG	**FORWARD**	**intra-exon:1B**
MGC1E-R1	TGCAGTGCTTGCTCAAAGGCAGCAAGAT	**REVERSE**	**intra-exon:1B**
MGC1E-R2	CTCTACCTGCCTCCAGGACTCATGAGT	**REVERSE**	**intra-exon:1B**
MGC2B-F1	ATGGAGTTTTGCTCTTGTTGCCCA	**FORWARD**	**intra-exon:2B**
MGC2B-F2	CAGTCTTGGCTCACCACAACCTC	**FORWARD**	**intra-exon:2B**
MGC223-F1	ATGCAAAGCTGCTTCCCTGAATC	**FORWARD**	**inter-exon:3-8**
MGC142-F1	ATGCTGACGCTGCGCACCAAGCTGTCA	**FORWARD**	**inter-exon:8-9**
MGC-EX6A-F1	CCAGCCACGTGACCTCTTCCA	**FORWARD**	**intra-exon:6A**
MGC-EX4-F1	AGAGCCCACCAGCTTCCGGGA	**FORWARD**	**intra-exon:4**
MGC-EX7-F1	ATGACTCTTCTACAAAAGTGGA	**FORWARD**	**intra-exon:7**
MGC-EX6-R1	CGCTGTGCAGGGACAGGTGGT	**REVERSE**	**intra-exon:6**
MGC-EX4-R1	CTGTCTTCAGGACCACCAGGTGT	**REVERSE**	**intra-exon:4**
MGC-EX2-R1	CTTTACCTCCTTCTCAATATAGT	**REVERSE**	**intra-exon:2**
**Full-length:**			
MGCF1	ATGGAAGAGGAGGGCAAGAA	**FORWARD**	**CCM2-A (Start codon)**
MGCR1	GCTGAGTCCTGGTCCATGCT	**REVERSE**	**HUMAN MGC**
MGCR2	TGCTGAGTCCTGGTCCAT	**REVERSE**	**HUMAN MGC**
MCGR3	TGCTGAGTCCTGGTCCATGCT	**REVERSE**	**HUMAN MGC**
MGCF-001	ATGCATAGTAGCTGTCGGCAGA	**FORWARD**	**CCM2-B (Start codon)**
MGC200-F1	ATGCATAGTAGCTGTCGGCAGAGGAGGA	**FORWARD**	**CCM2-B (Start codon)**

Suppl. Table 2

RT-PCR Code	Primer	Sequence	Product Size (bp)	Representig Isoform(s)	Anneling Temp (C)
**A-100**	MGC-EXB1-2-F1	GGCAAGAAGCCTGGAATTGT	**98**	**100, 103, 104,115, 116, 117**	**65**
	**MGC-EX2-R1**	CGCCTCTCTGTCACCTTCTC		**118, 119, 402, 404, 602**	
**A-101**	MGC-EXB1-3-F1	GGGCAAGAAGTATTTAGGTCAG	**103**	**101, 102, 120, 401, 601, 603**	**60**
	MGC-EXB3-4-R1	GTGGGCTCTCTTTGCATTGT			
**A-102**	MGC-EXB1-3-F1	GGGCAAGAAGTATTTAGGTCAG	**291**	**102**	**65**
	MGC-EXB4-7-R1	GTAGAAGAGTCATCTGTCTTCAGGA			
**B-213**	MGC-EXB2-3-F1	GGAGGTAAAGTATTTAGGTCAG	**291**	**117, 203, 404**	**65**
	MGC-EXB4-7-R1	GTAGAAGAGTCATCTGTCTTCAGGA			
**A-117**	MGC-EXB4-7-R1	GTAGAAGAGTCATCTGTCTTCAGGA	**148**	**102, 117, 203, 404, 210**	**60**
	**MGC-EX4-F3**	TCAGCCTGTCTGCGTACAAC			
**B-204**	MGC-EXB1A-2-F1	GGAGAATGAGCCTGGAATTG	**193**	**204**	**60**
	MGC-EXB2-4-R1	GTGGGCTCTCTTTACCTCCTT			
**B-209**	MGC-EXB1A-8-F1	GAGAATGAGCTGCTTCCCTGA	**146**	**209**	**65**
	**MGC-EX9-R1**	CCTGTGATGACAGCTTGGTG			
**B-210**	MGC-EXB1A-3-F1	GGAGAATGAGTATTTAGGTCAG	**291**	**210**	**55**
	MGC-EXB4-7-R1	GTAGAAGAGTCATCTGTCTTCAGGA			
**B-213**	MGC-EXB6-7as01-F1	TGCACAGCGATGACTCTTCT	**89**	**213**	**60**
	MGC-EX10as04-R1	GCCAAAGCTGTCAGTGATGA			
**A-401**	MGC-EXB1-3-F1	GGGCAAGAAGTATTTAGGTCAG	**138**	**401**	**60**
	MGC-EXB-4as01-10as01-R1	ATGTGGTGCTCCTGAGTCAA			
**A-116**	MGC-EXB1-2-F1	GGCAAGAAGCCTGGAATTGT	**193**	**116**	**60**
	MGC-EXB2-6-R2	CGCAGCGACCTTTACCTCCTT			
**B-200**	MGC-EXB1A-2-F1	GGAGAATGAGCCTGGAATTG	**120**	**200, 203, 204, 206**	**65**
	**MGC-EX2-R2**	AACACCACAGTGTGCAGAGG		**207, 208,213, 216,**	
**B-201**	MGC-EXB3-4-R1	GTGGGCTCTCTTTGCATTGT	**103**	**201, 202, 210, 214**	**60**
	MGC-EXB1A-3-F2	CCATGGAGAATGAGTATTTAGGTCAG			
**A-402**	MGC-EXB3-4as02-F2	CAAAGAGAGCCCACCAGCTT	**104**	**402**	**65**
	MGC-EXB4as02-10as02-R2	CTCATCCCCGTCCCTCCA			
**A-404**	MGC-EXB9-10as04-F2	GTTCCTGCTGCTTGGTCTGA	**87**	**404**	**65**
	MGC-EXB10as04-10as05-R2	CCATCATGGCCAATGGTCT			
**AB-6A**	MGC-EXB6A-7-F2	CTCCCTATTTCAAGATGACTCTTCTAC	**103**	**EX6A**	**60**
	**MGC-EX8-R2**	ACCACCCACATCCACAGATT			
**CCM3**	PDCD102F1	ATGAGGATGACAATGGAAGAGATGAAG	**307**	**PDCD10**	**65**
	PDCD101R2	GGTCTTGGAATTCTGGCTCTGGTCGTTC			
**V5**	EX10V5-F1	CCAGCACTTCGAGAACTTCC	**265**	**V5**	**65**
	EX10V5-R1	ATCTGCAGAATTGCCCTTtg			
**Neo**	Neo-F1	CTGAATGAACTGCAGGACGA	**172**	**Neo**	**65**
	Neo-R1	ATACTTTCTCGGCAGGAGCA			

Suppl. Table 3

Isoform identifier	Length (aa)	PTB domain	Domain-2	Domain-3	Domain-4
CCM2-100*	444	61-221: PTB (161aa)	244-361:Protein of unknown function (DUF1722) (118 aa)	SSL2: DNA or RNA helicases of superfamily II	
CCM2-101	386	1-163:PTB (162aa)	186-303:Protein of unknown function (DUF1722) (118 aa)	SSL2: DNA or RNA helicases of superfamily II	
CCM2-102	295	1-143:PTB (143aa)	95-212:Protein of unknown function (DUF1722) (118 aa)	SSL2: DNA or RNA helicases of superfamily II	
CCM2-107	251	NO PTB	51-168:Protein of unknown function (DUF1722) (118 aa)	SSL2: DNA or RNA helicases of superfamily II	
CCM2-109	142	NO PTB	1-59:Protein of unknown function (DUF1722) (59 aa)	SSL2: DNA or RNA helicases of superfamily II	Excinuclease ABC subunit A
CCM2-116	309	NO PTB	1-59:Protein of unknown function (DUF1722) (59 aa)	SSL2: DNA or RNA helicases of superfamily II	Flagellar hook-associated protein L
CCM2-117	353	60-201: PTB (142aa)	153-270:Protein of unknown function (DUF1722) (118 aa)	SSL2: DNA or RNA helicases of superfamily II	
CCM2-402	153	NO PTB	4-153:N5-glutamine S-adenosyl-L-methionine-dependent methyltransferase		
CCM2-404	334	60-201: PTB (142aa)		SSL2: DNA or RNA helicases of superfamily II	
CCM2-600	485	60-221: PTB (162aa)	285-402:Protein of unknown function (DUF1722)	SSL2: DNA or RNA helicases of superfamily II	
CCM2-601	427	1-163: PTB (163aa)	227-344:Protein of unknown function (DUF1722)	SSL2: DNA or RNA helicases of superfamily II	
CCM2-602	188	60-188: PTB (129aa)	44-188: Type 1 glutamine amidotransferase (GATase1)-like domain		
CCM2-200	465	81-242: PTB (162aa)	265-382:Protein of unknown function (DUF1722) (118 aa)	SSL2: DNA or RNA helicases of superfamily II	
CCM2-201	407	22-184: PTB (163aa)	207-324:Protein of unknown function (DUF1722) (118 aa)	SSL2: DNA or RNA helicases of superfamily II	1-238: Thiamin transporter substrate binding subunit
CCM2-203	374	81-222: PTB (142aa)	174-291:Protein of unknown function (DUF1722) (118 aa)	SSL2: DNA or RNA helicases of superfamily II	
CCM2-204	437	52-214: PTB (163aa)	237-354:Protein of unknown function (DUF1722) (118 aa)	SSL2: DNA or RNA helicases of superfamily II	1-187:tRNA pseudouridine synthase C
CCM2-206	506	81-242: PTB (162aa)	306-423:Protein of unknown function (DUF1722) (118 aa)	SSL2: DNA or RNA helicases of superfamily II	
CCM2-210	316	22-164: PTB (143aa)	116-233:Protein of unknown function (DUF1722) (118 aa)	SSL2: DNA or RNA helicases of superfamily II	
CCM2-212	272	NO PTB	72-189:Protein of unknown function (DUF1722) (118 aa)	SSL2: DNA or RNA helicases of superfamily II	
CCM2-213	354	81-242: PTB (162aa)	1-296:tbpA (thiamin transporter substrate binding subunit)(296aa)		
CCM2-216	415	81-242: PTB (162aa)	215-333:Protein of unknown function (DUF1722) (119 aa)	SSL2: DNA or RNA helicases of superfamily II	PRK07168

Suppl. Table 4

Motif	Sequence	Length (aa)	***Domains***
CCM2-Exon6<br>***Isoform***	**VAAEELCCLLGQVFQVVYTE*STIDFLDRAIFDGAST*PTHHLSLHS**<br>*CCM2-107, CCCM2-116, CCM2-212*	**45**	**No putative conserved domain**<br>**No relatipnship with PTB 1**
CCM2-Exon6A<br>***Isoform***	**ASHVTSSTVTSSFDCQLPLHFRTLAITLSPPKFSRVISLFQ**<br>*CCM2-600, CCM2-601, CCM2-606, CCM2-607,CCM2-206*	**41**	**No putative conserved domain**<br>**No relatipnship with PTB 1**
CCM2-Exon6B<br>***Isoform***	**DHGQTHGKLLRLPWEASWQWWSSGRWGRMEGTPELSLPAWPTQHFQE**<br>*CCM2-610*	**47**	**No putative conserved domain**<br>**No relatipnship with PTB 1**

Suppl. Fig. 1A Brain

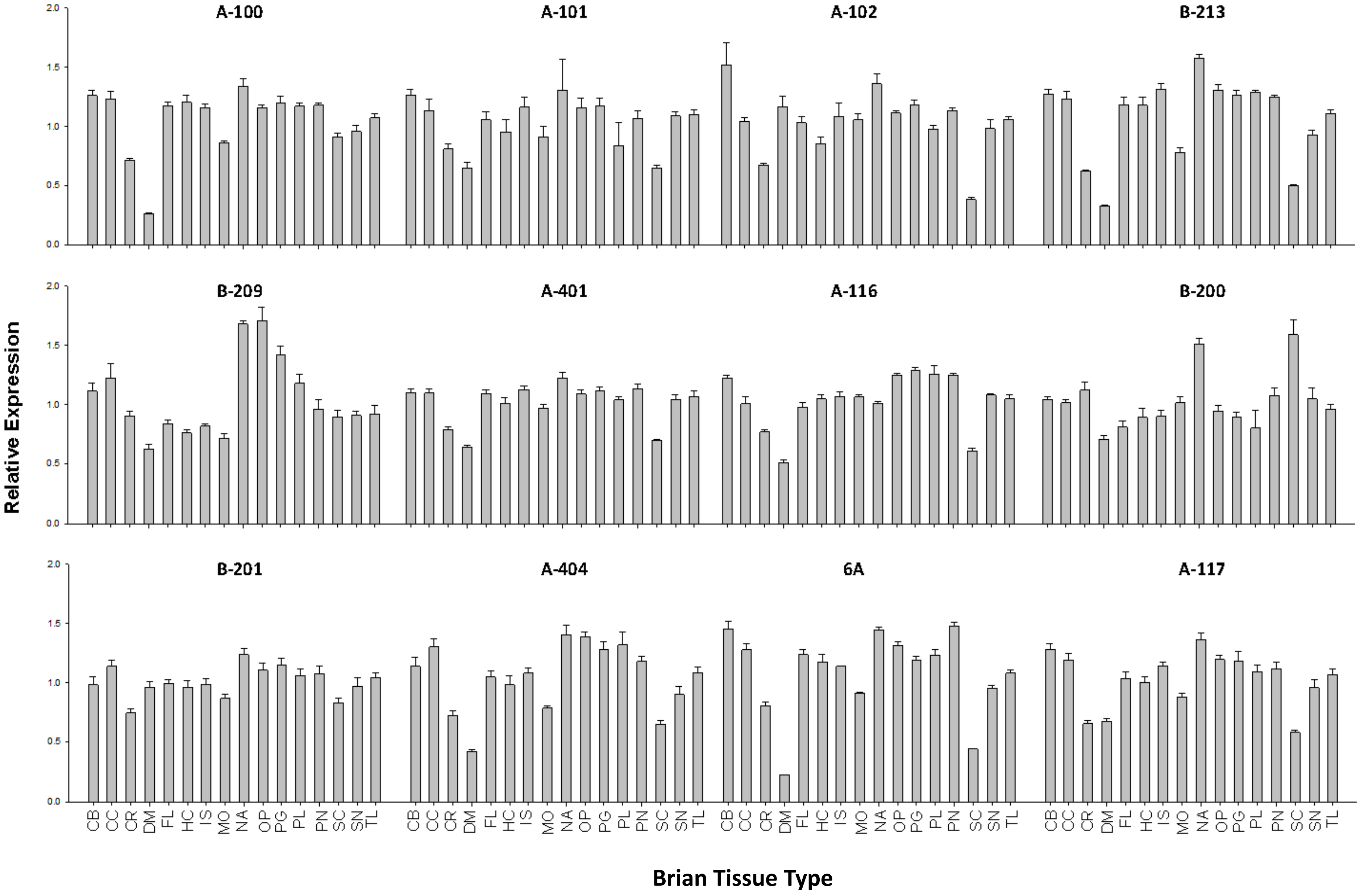

Suppl. Fig. 1B Cell

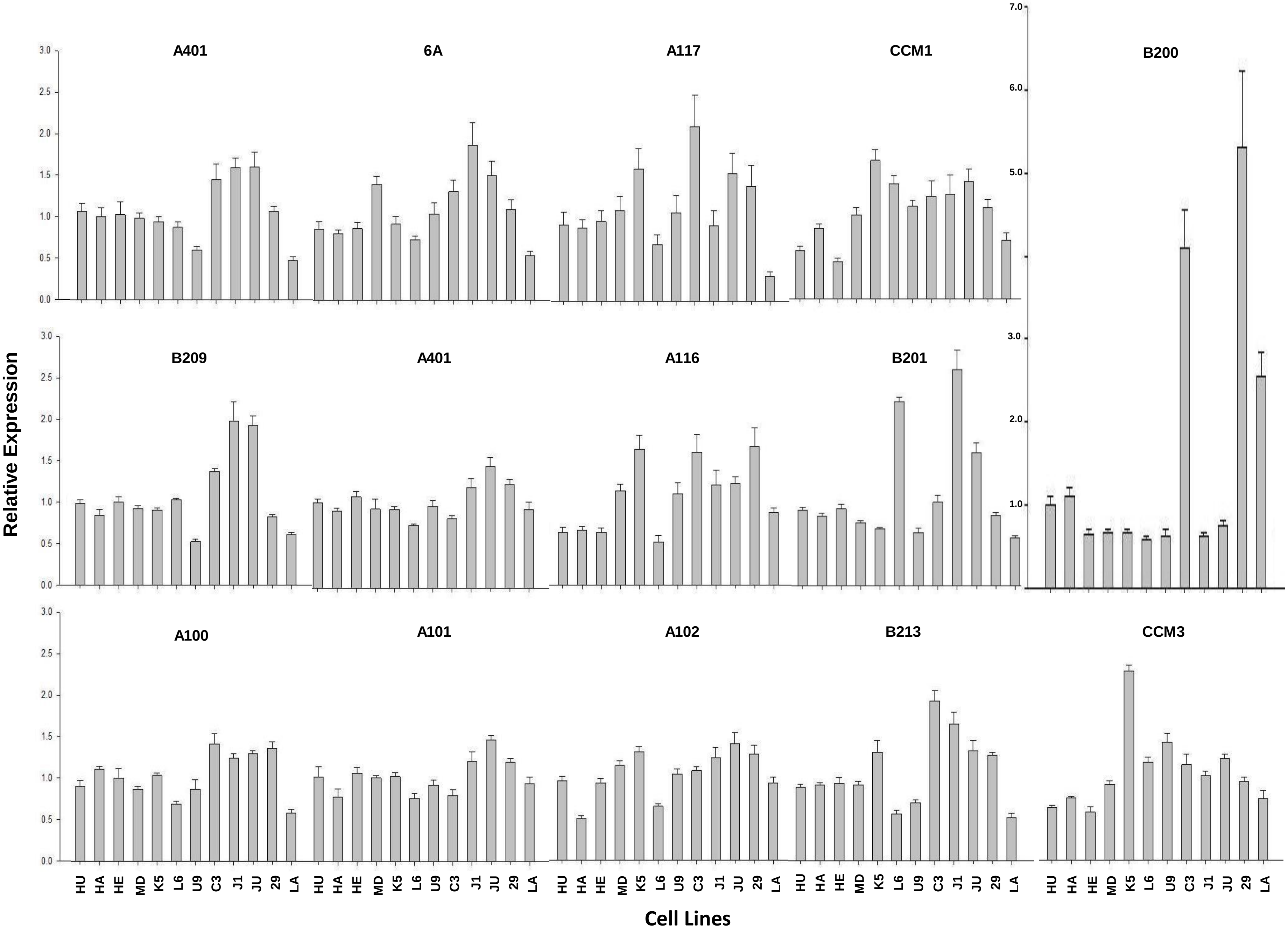

Suppl. Fig. 2A HeLa

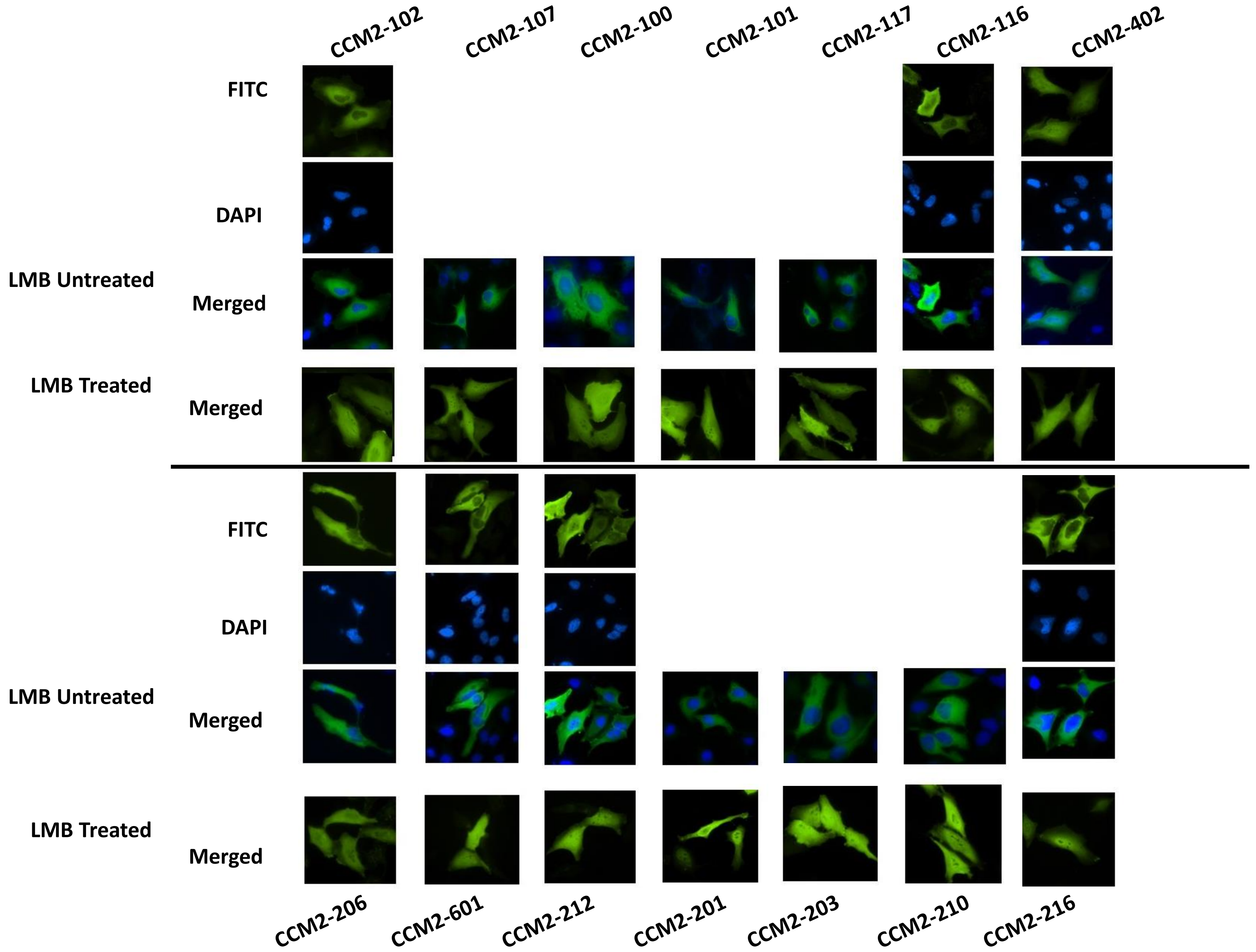

Suppl. Fig. 2B HUVEC

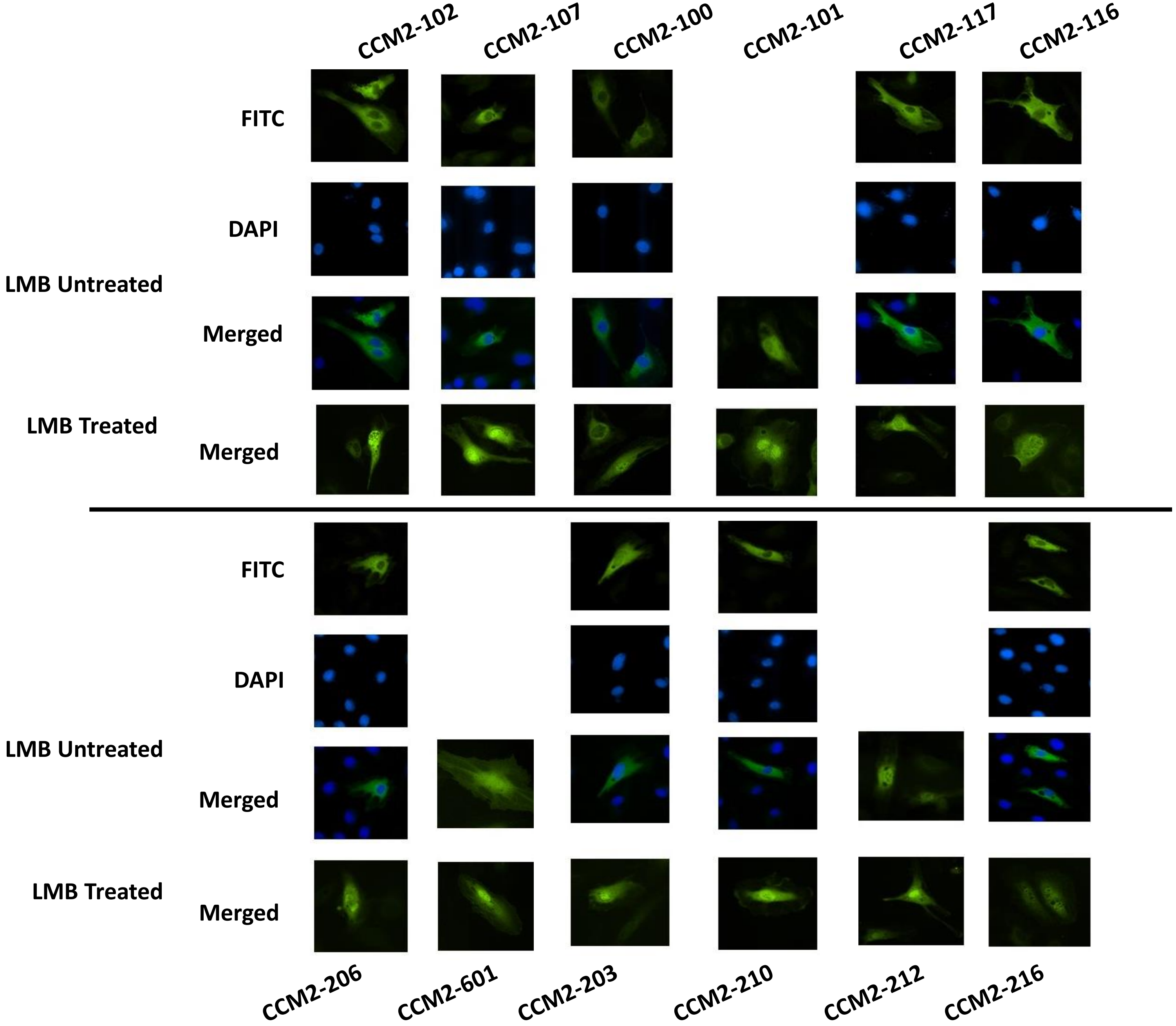

Suppl. Fig. 2C HMVEC

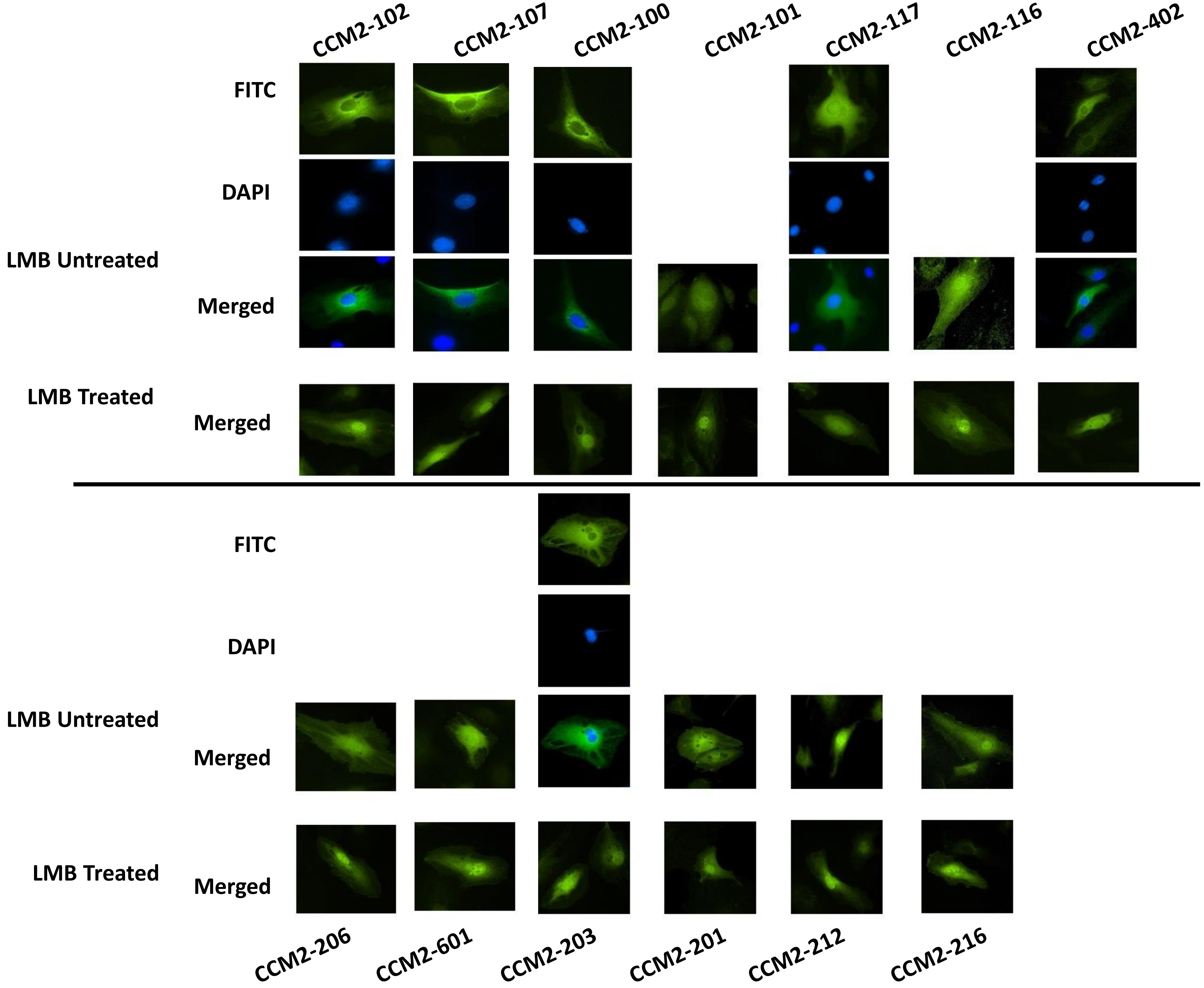

Suppl. Fig. 2D hCMEC/3D

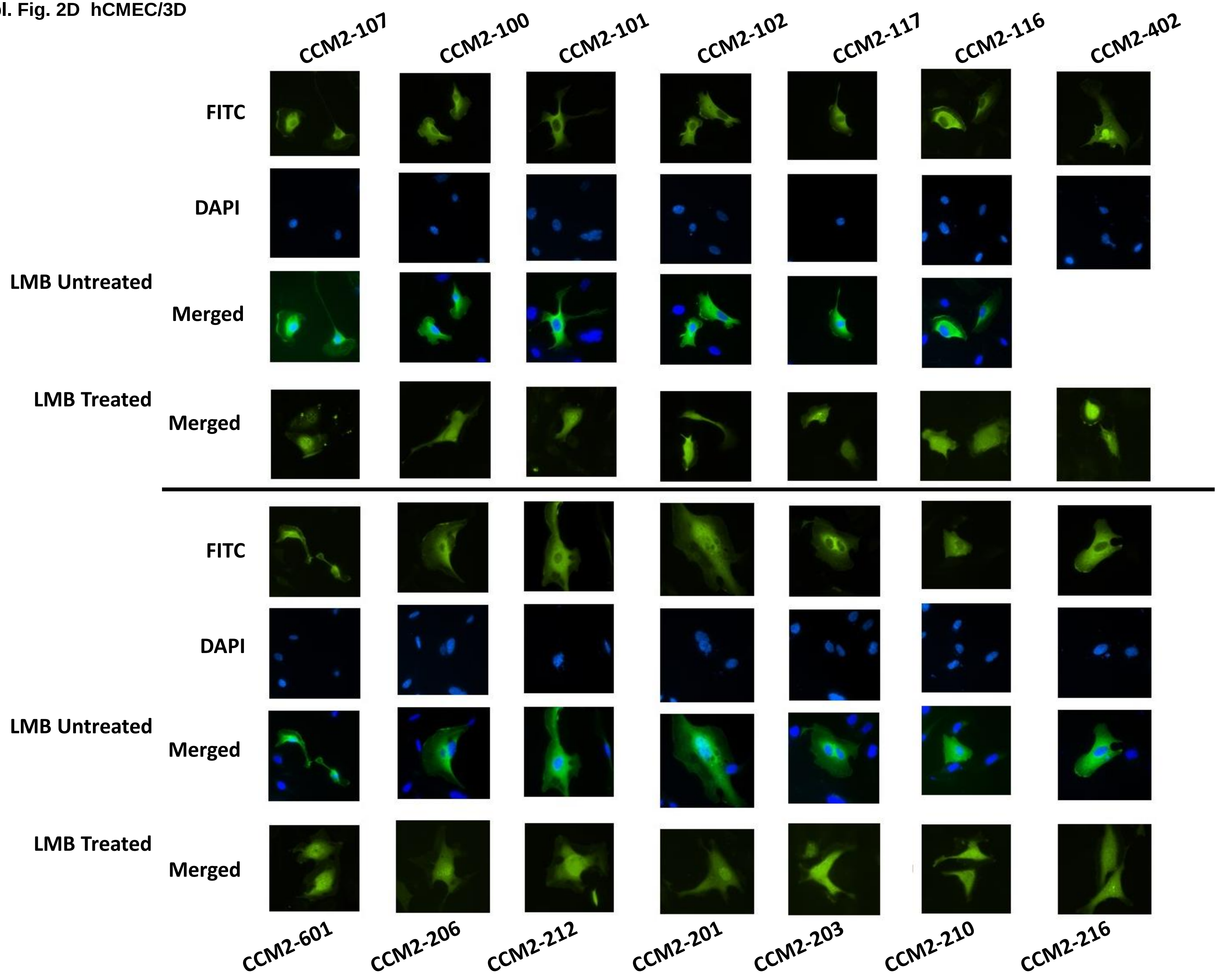

3B1

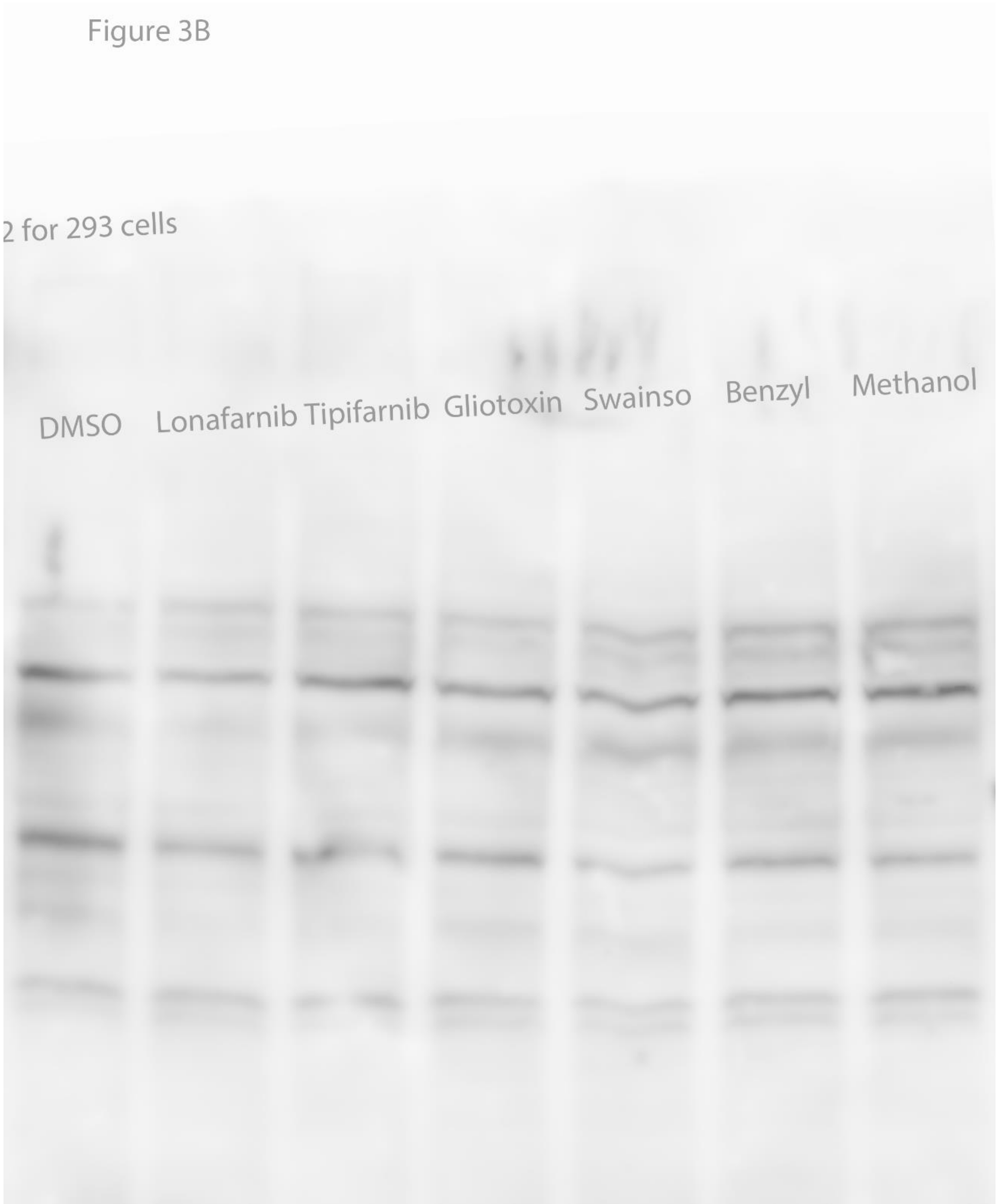
Figure 3B
2 for 293 cells
DMSO
Lonafarnib
Tipifarnib
Gliotoxin
Swainso
Benzyl
Methanol

3B2

Fig.3B. Actinin control

for 293 cells

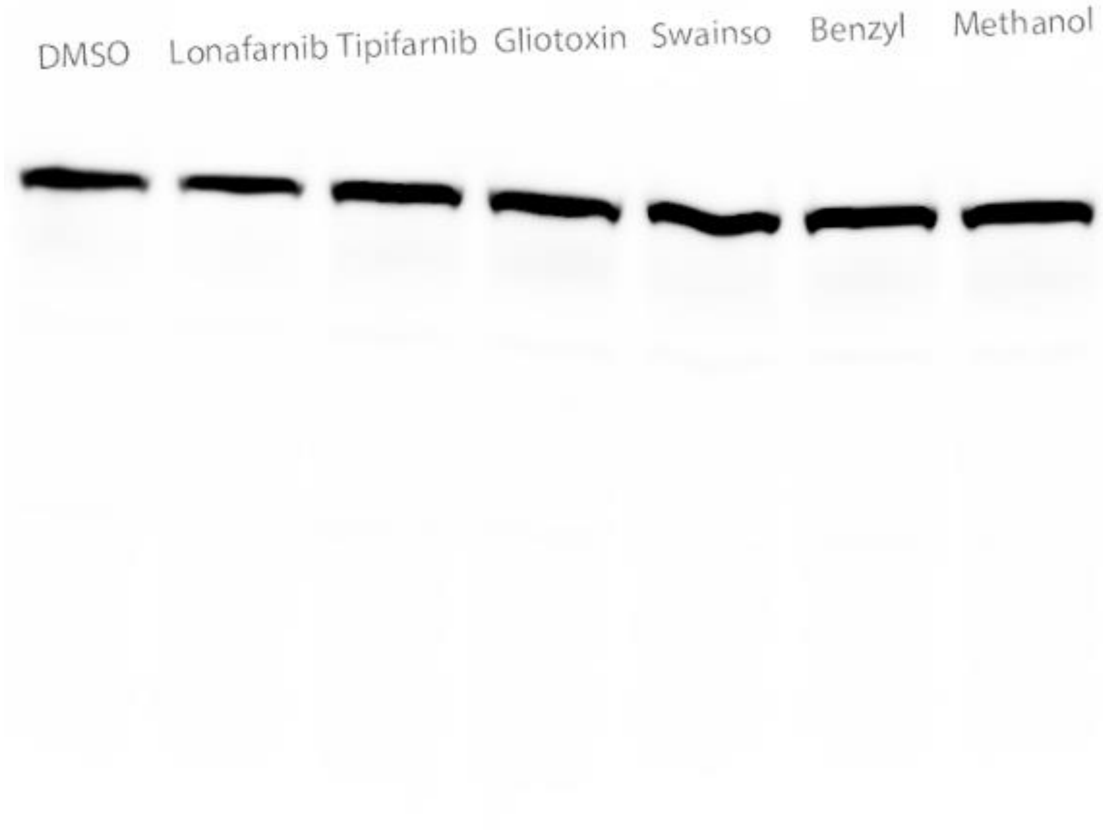

3C1

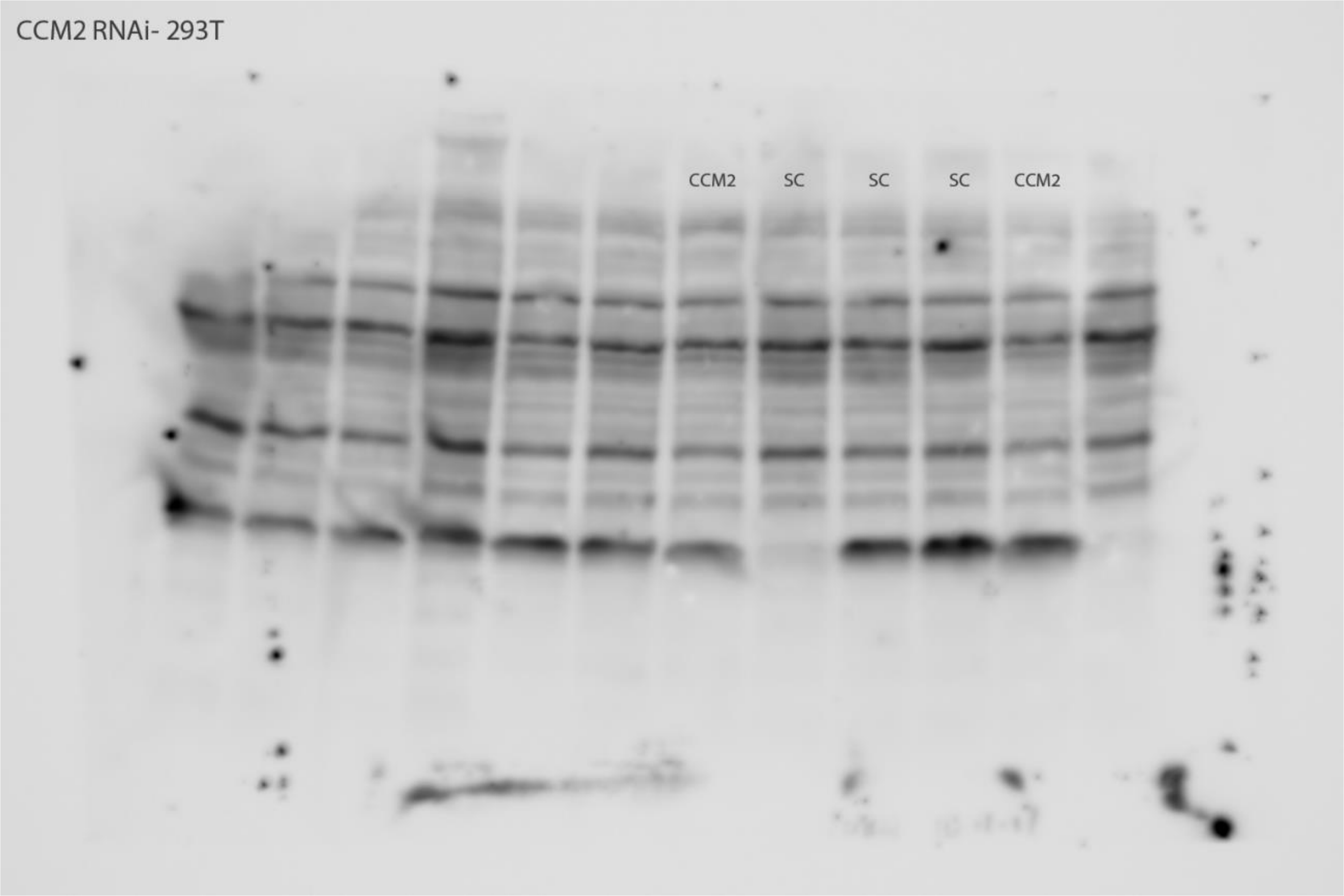

3C2

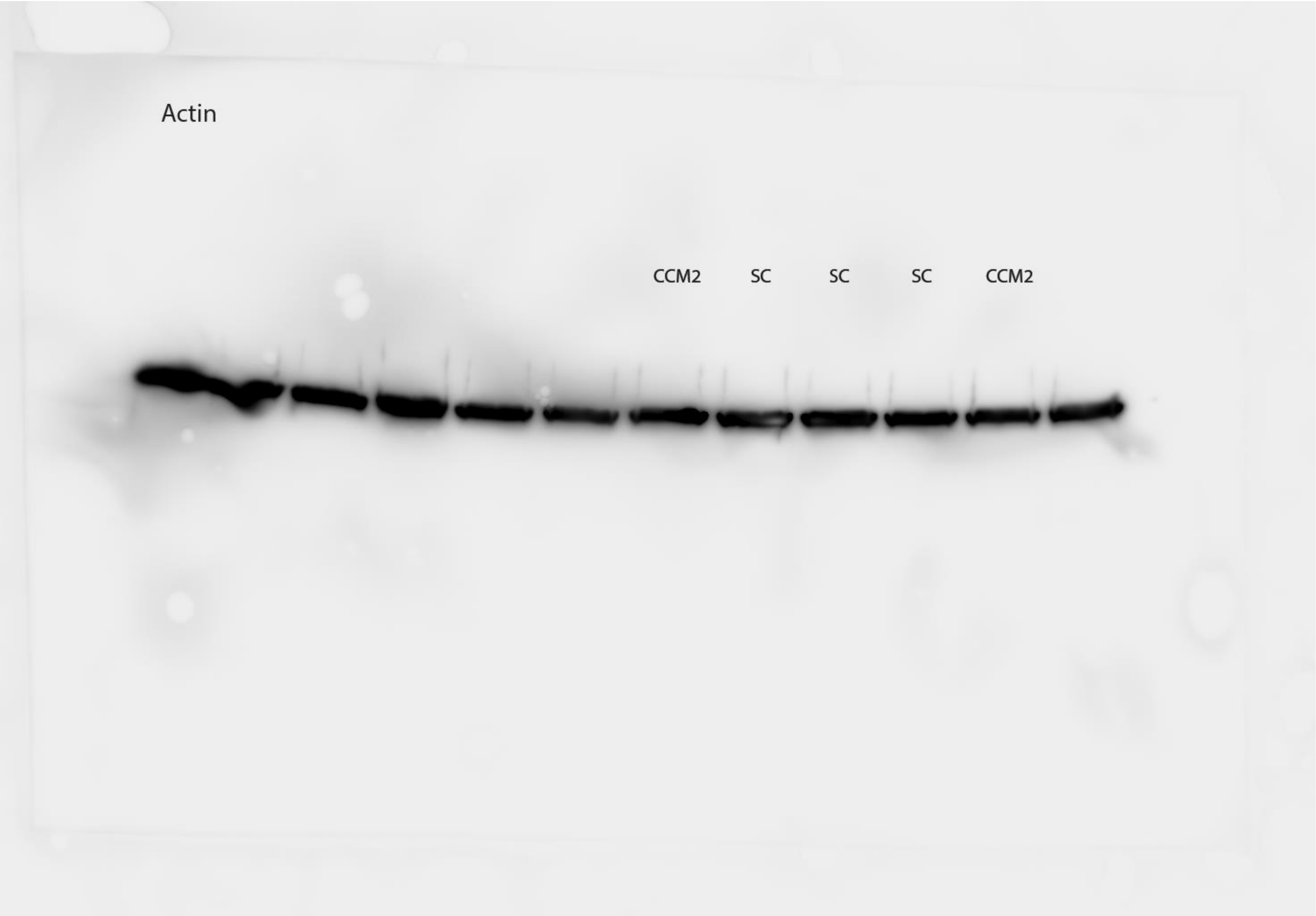

6A

Pre-IP

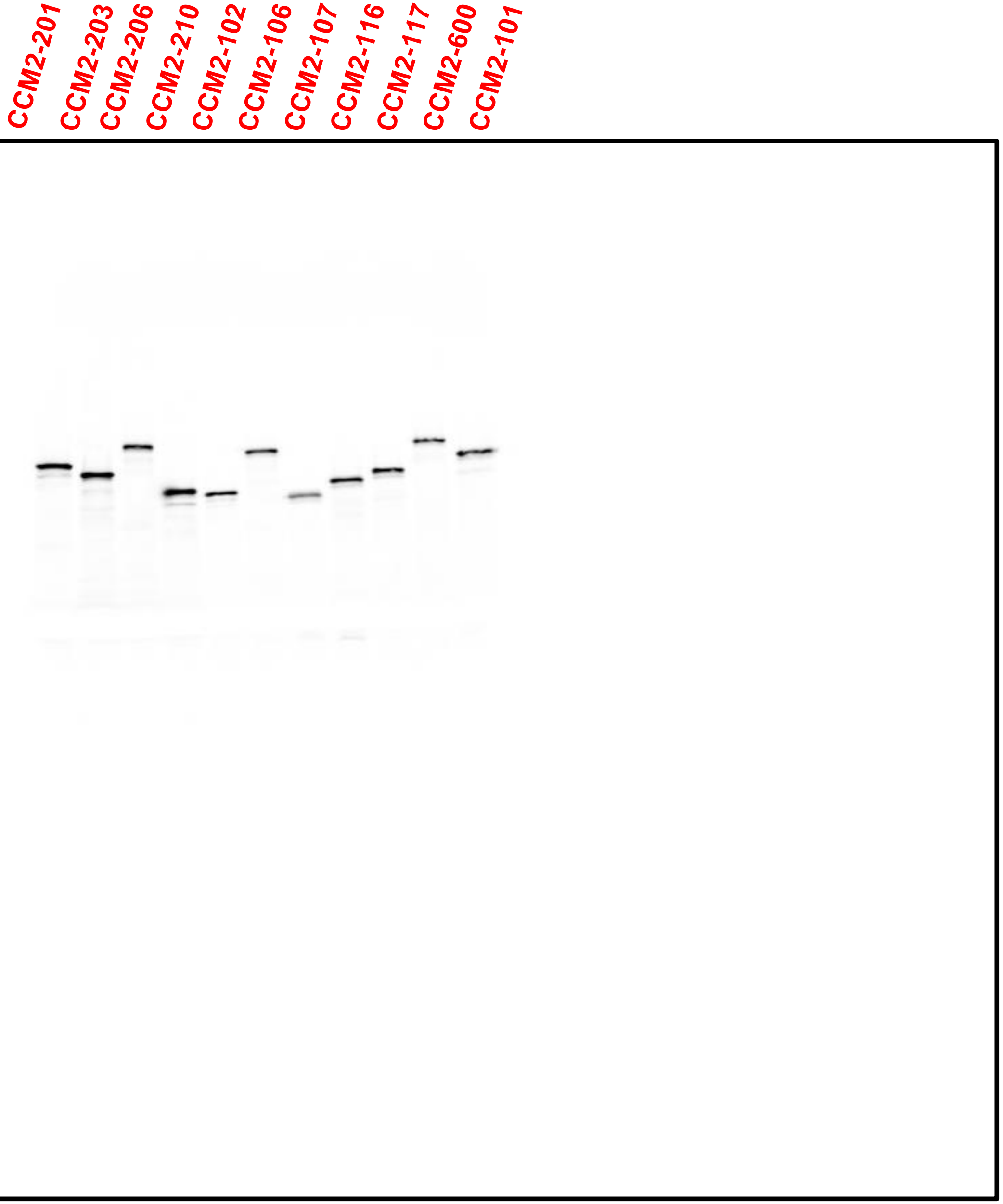
CCM2-201
CCM2-203
CCM2-206
CCM2-210
CCM2-102
CCM2-106
CCM2-107
CCM2-116
CCM2-117
CCM2-600
CCM2-101

6A1

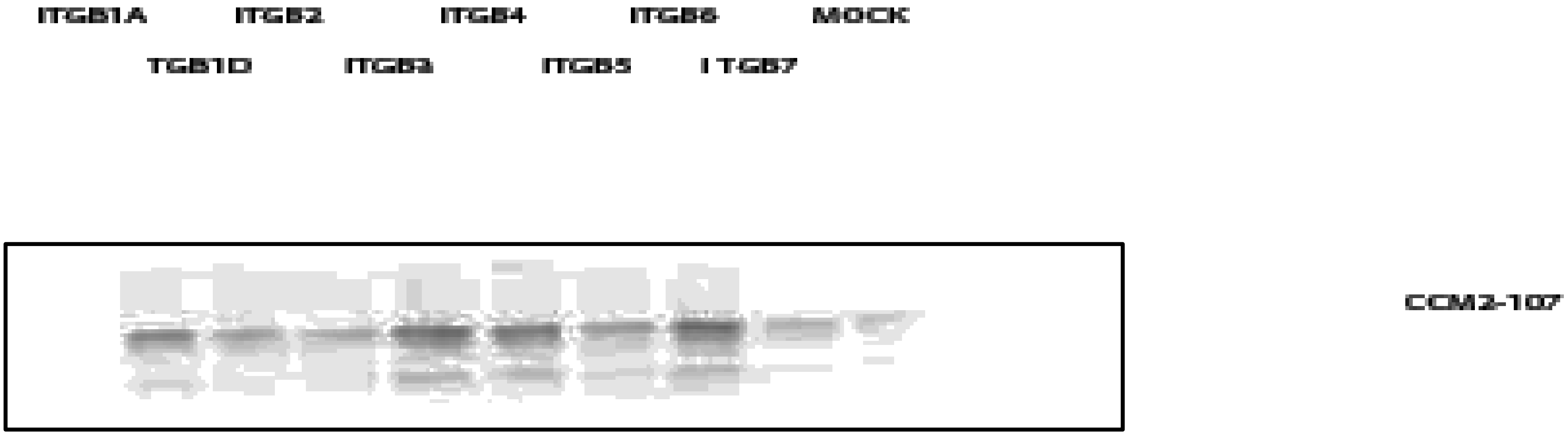
ITGB1A
TGB1D
ITGB2
ITGB3
ITGB4
ITGB5
ITGB6
I TGB7
MOCK
CCM2-107

6A2

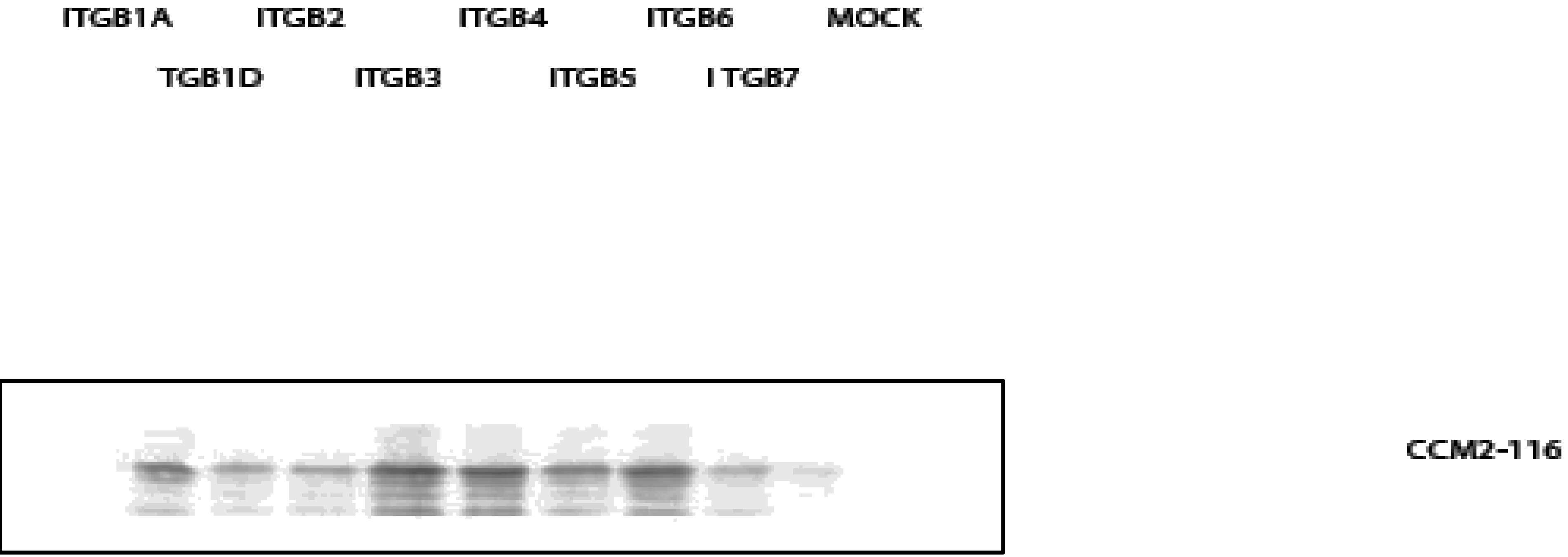
ITGB1A
ITGB2
ITGB4
ITGB6
MOCK
TGB1D
ITGB3
ITGB5
I TGB7
CCM2-116

6A3

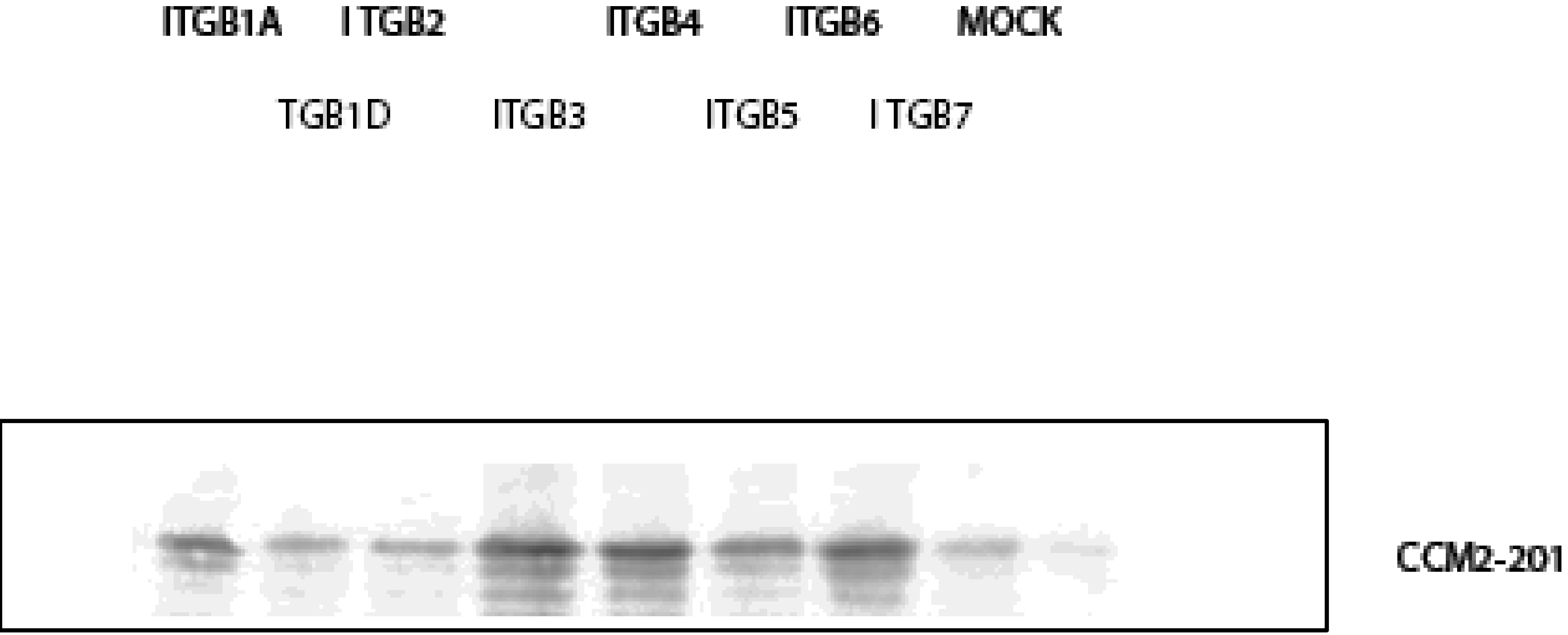
ITGB1A
ITGB2
ITGB4
ITGB6
MOCK
TGB1D
ITGB3
ITGB5
ITGB7
CCM2-201

6A4

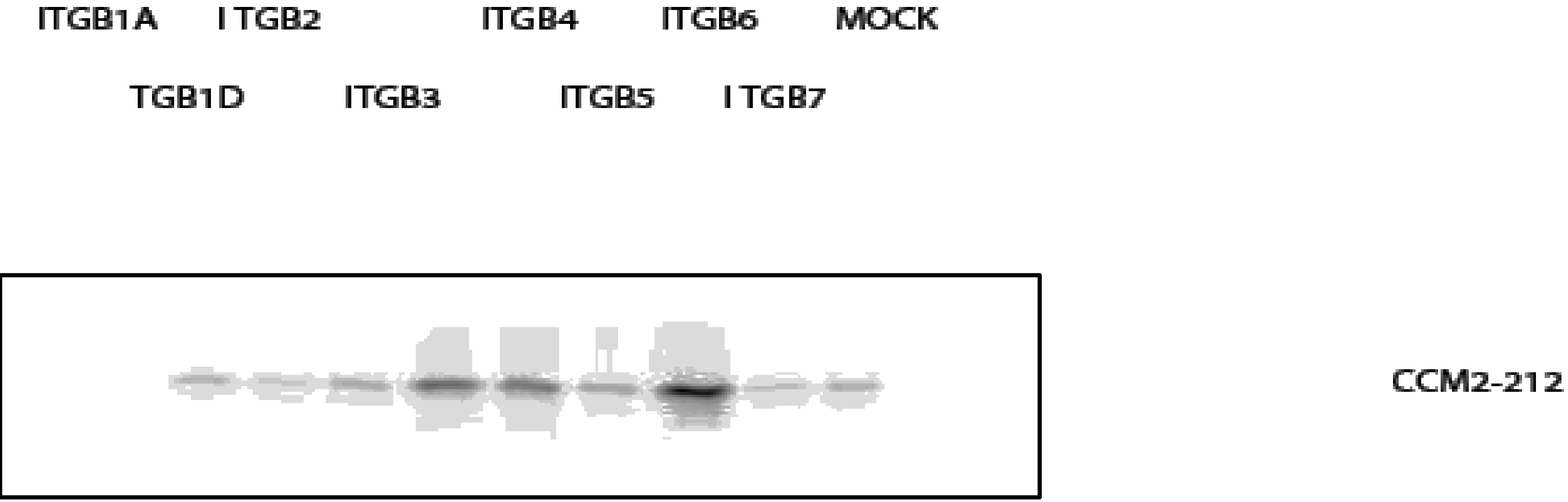
ITGB1A
I TGB2
ITGB4
ITGB6
MOCK
TGB1D
ITGB3
ITGB5
I TGB7
CCM2-212

Loading control

6B1

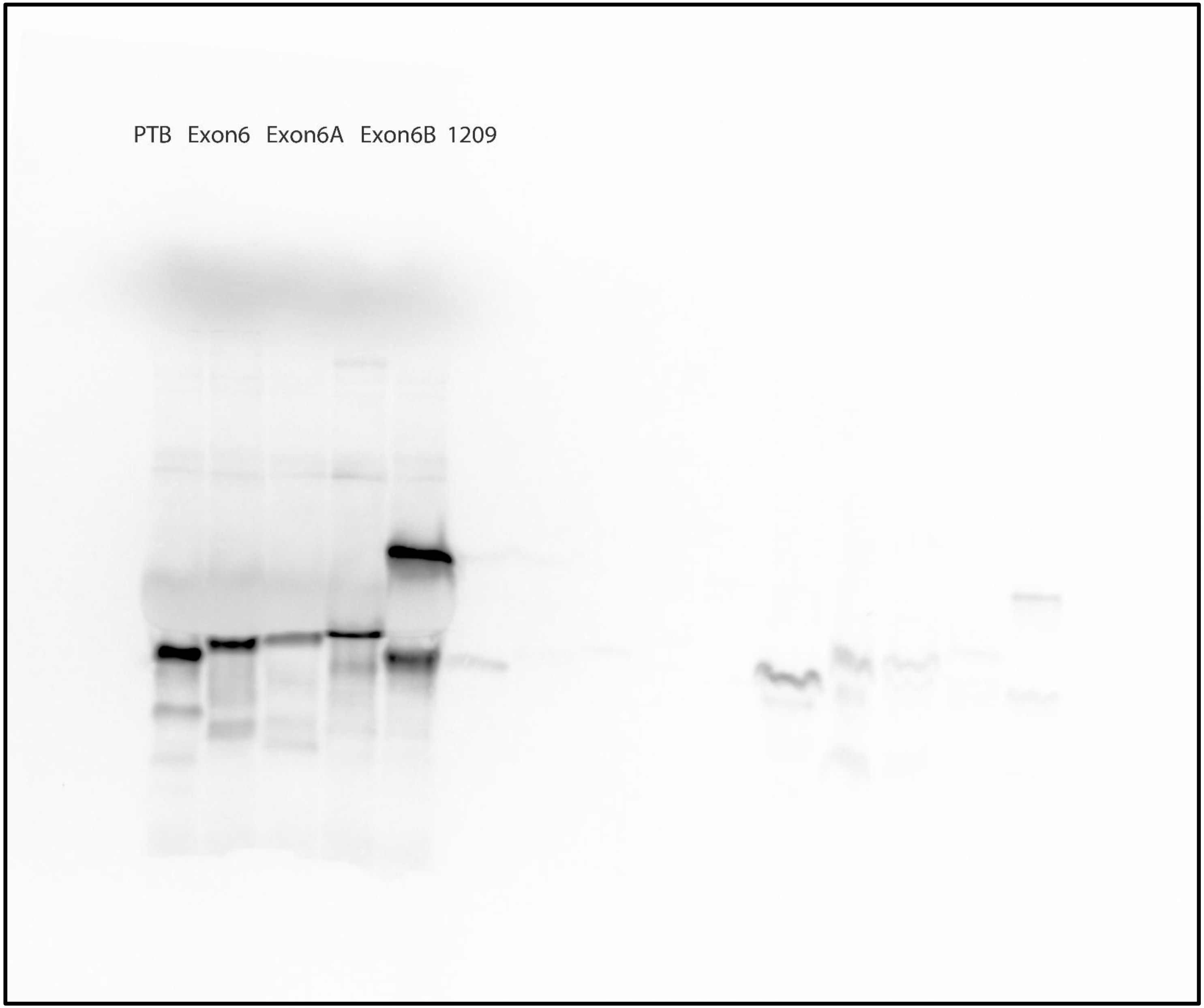

6B2

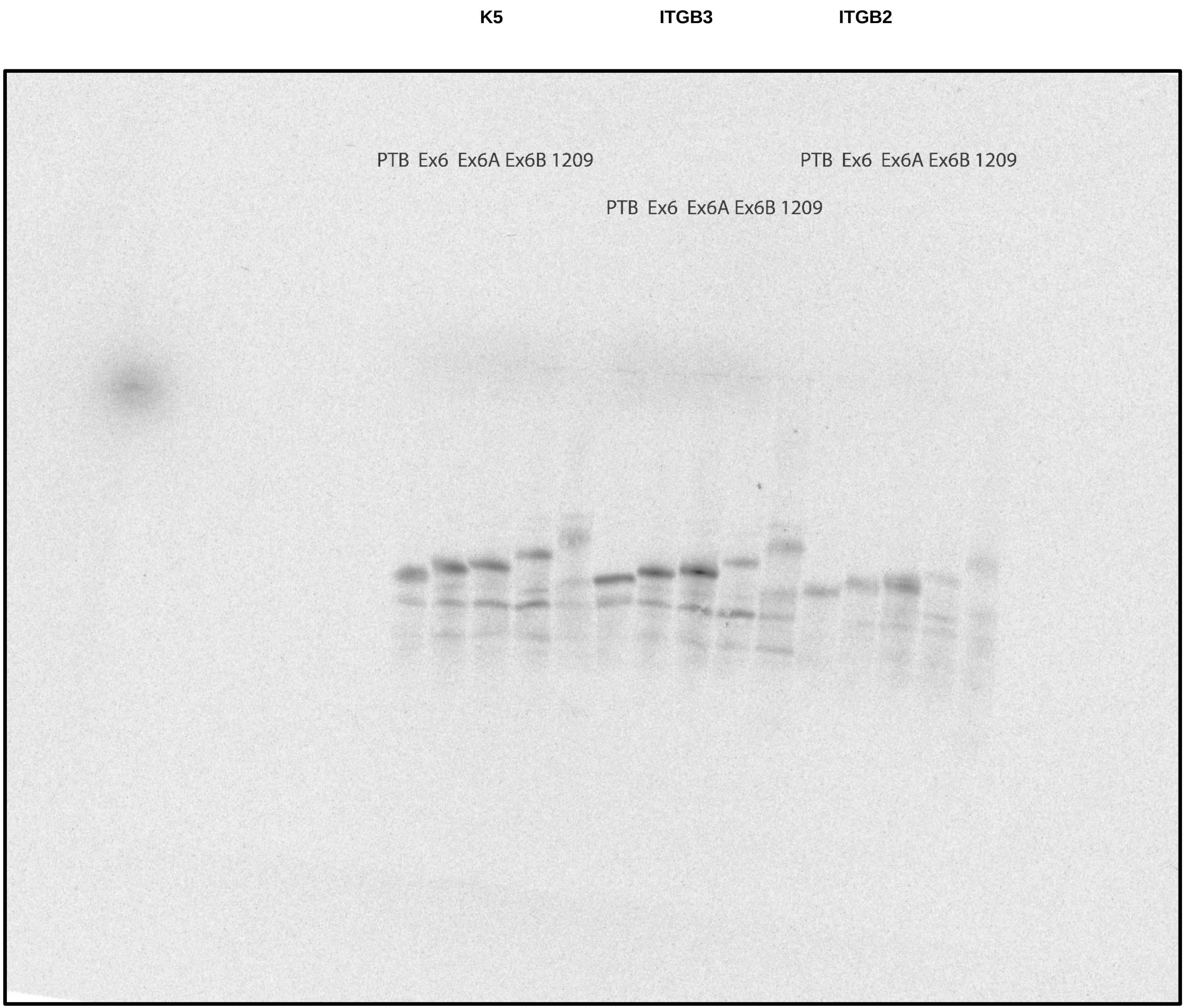
K5
ITGB3
ITGB2
PTB Ex6 Ex6A Ex6B 1209
PTB Ex6 Ex6A Ex6B 1209
PTB Ex6 Ex6A Ex6B 1209

6B3

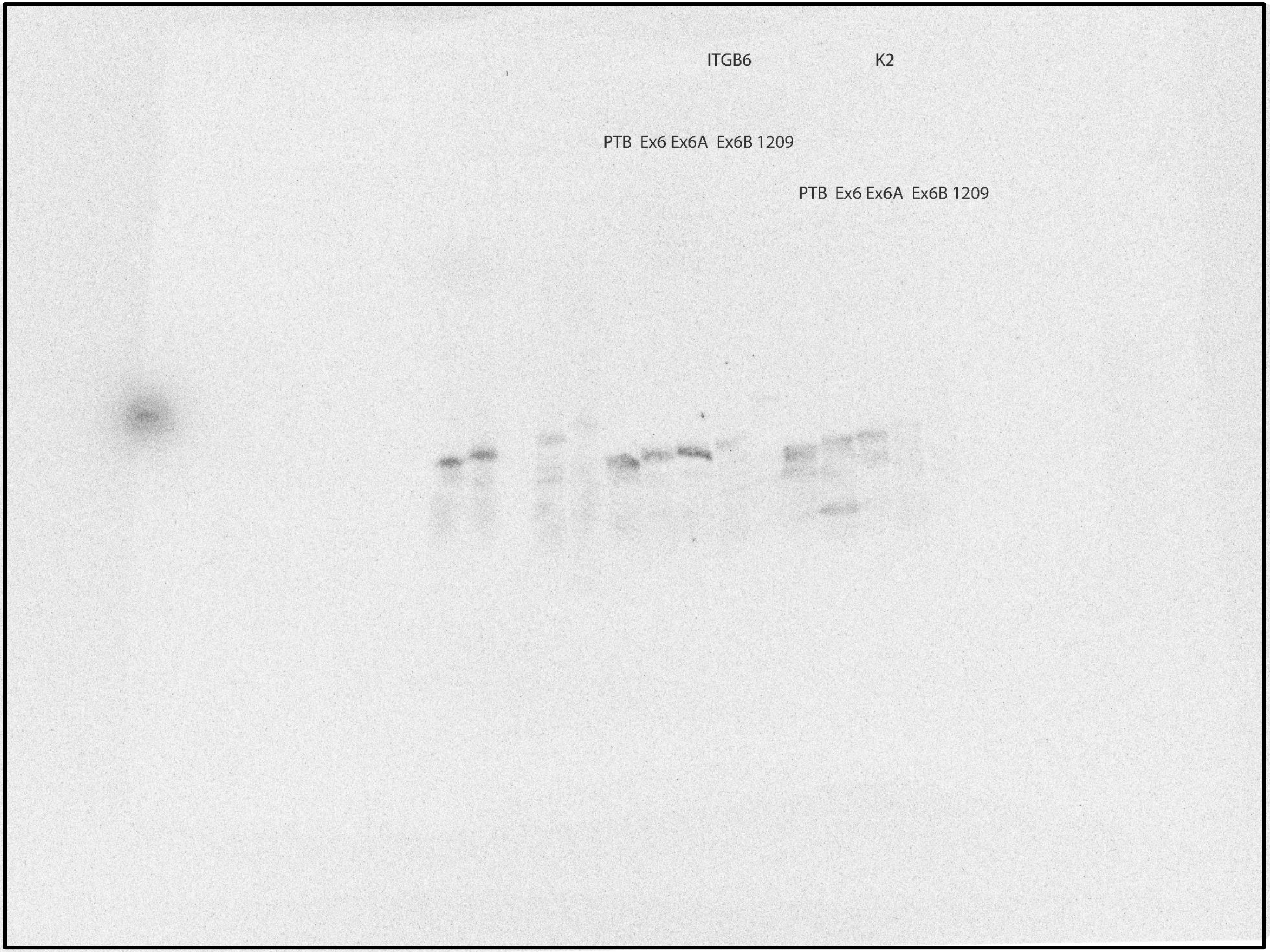

6B4

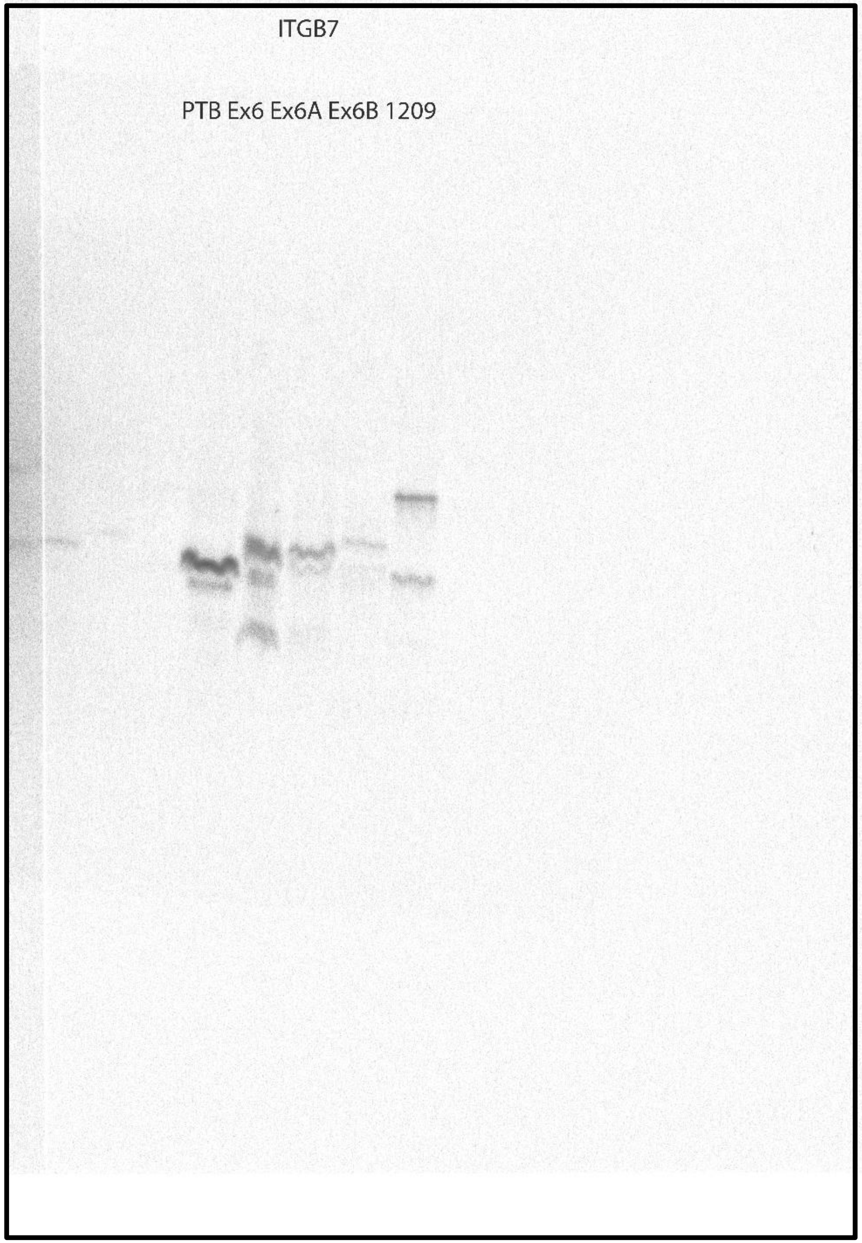

6C1

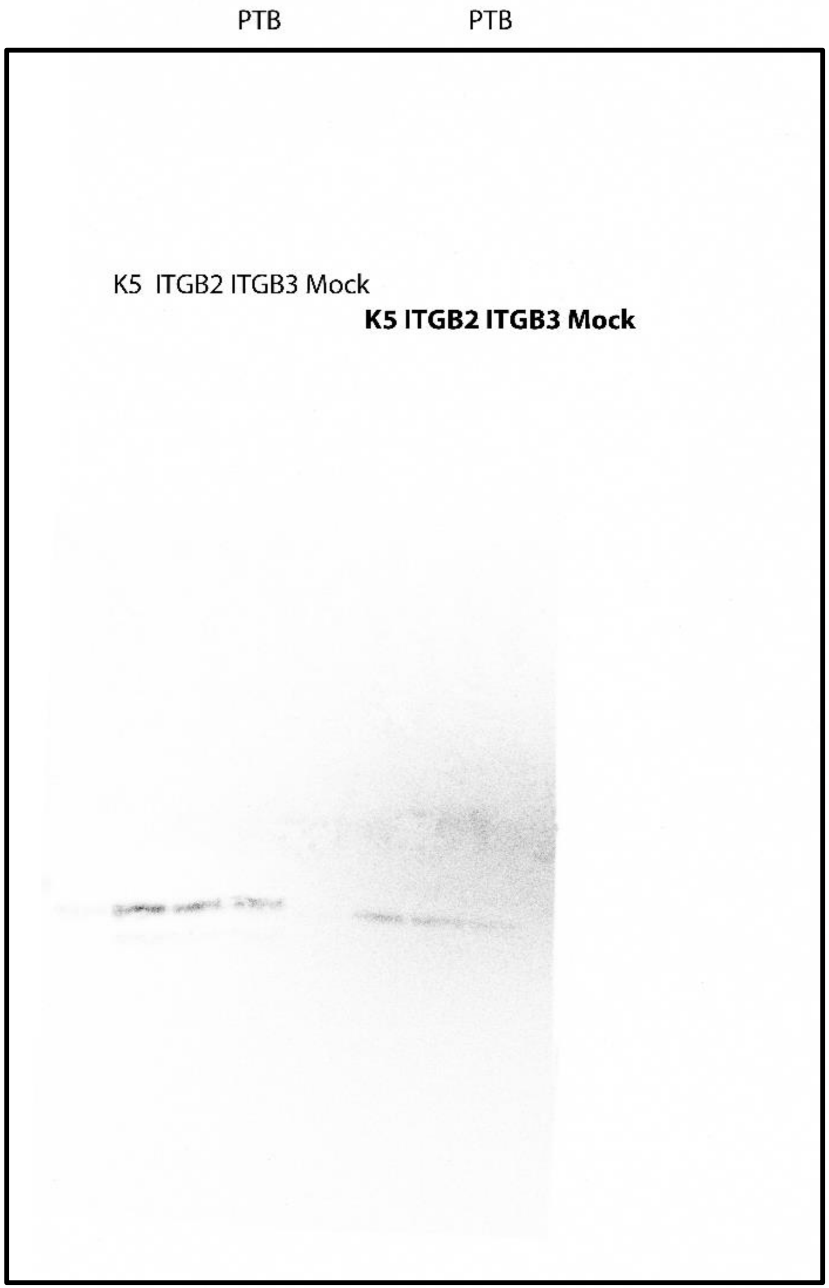

6C2-3

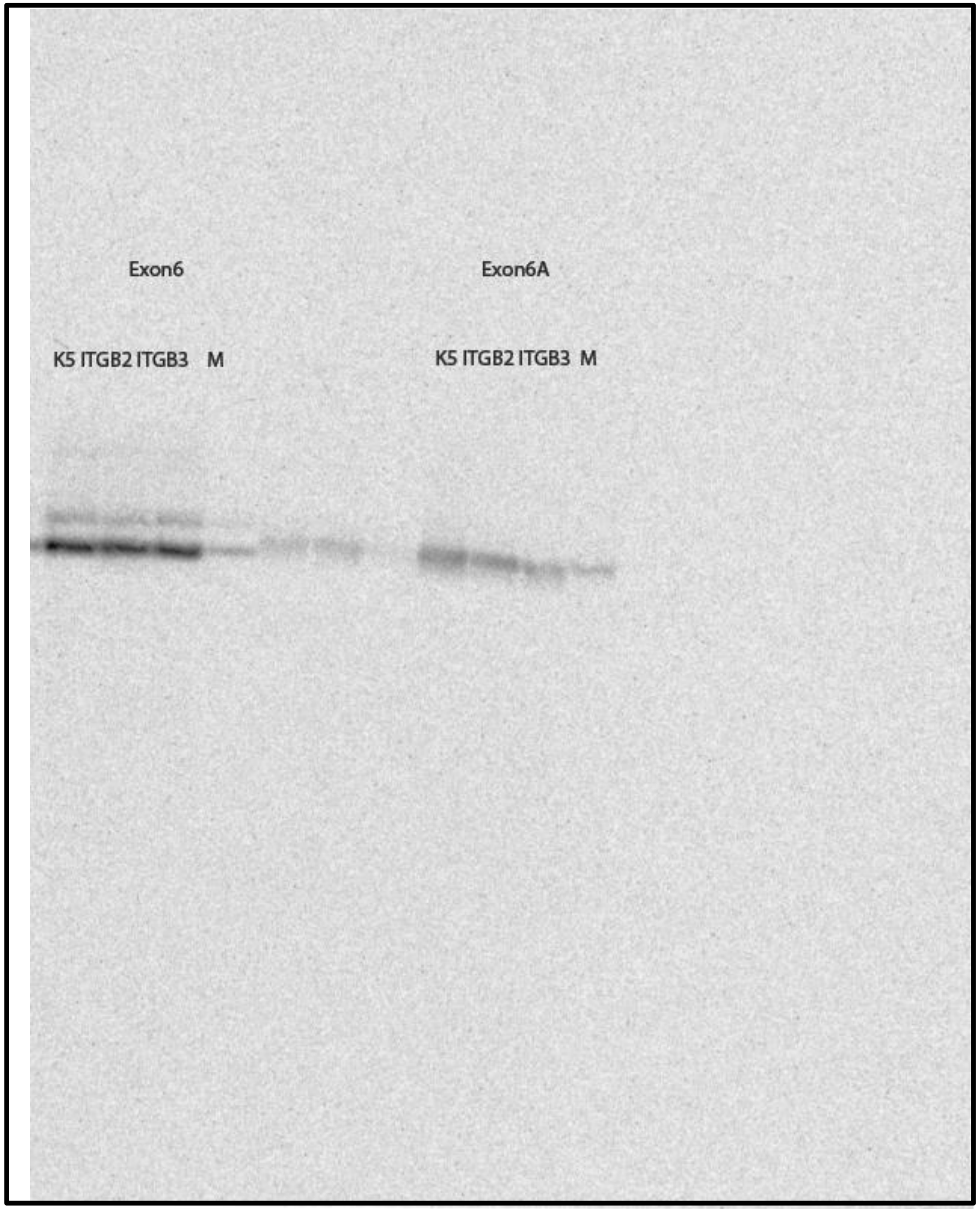

Supplement: Supplementary file 1 — Supplemental information [file 41598_2019_52386_MOESM1_ESM.pdf]
